# The HUNT study: A population-based cohort for genetic research

**DOI:** 10.1016/j.xgen.2022.100193

**Published:** 2022-10-12

**Authors:** Ben M. Brumpton, Sarah Graham, Ida Surakka, Anne Heidi Skogholt, Mari Løset, Lars G. Fritsche, Brooke Wolford, Wei Zhou, Jonas Bille Nielsen, Oddgeir L. Holmen, Maiken E. Gabrielsen, Laurent Thomas, Laxmi Bhatta, Humaira Rasheed, He Zhang, Hyun Min Kang, Whitney Hornsby, Marta Riise Moksnes, Eivind Coward, Mads Melbye, Guro F. Giskeødegård, Jørn Fenstad, Steinar Krokstad, Marit Næss, Arnulf Langhammer, Michael Boehnke, Gonçalo R. Abecasis, Bjørn Olav Åsvold, Kristian Hveem, Cristen J. Willer

**Affiliations:** 1 K.G. Jebsen Center for Genetic Epidemiology, Department of Public Health and Nursing, NTNU, Norwegian University of Science and Technology, Trondheim 7030, Norway; 2 HUNT Research Centre, Department of Public Health and Nursing, NTNU, Norwegian University of Science and Technology, Levanger 7600, Norway; 3 Clinic of Medicine, St. Olavs Hospital, Trondheim University Hospital, Trondheim 7030, Norway; 4 Department of Internal Medicine, Division of Cardiology, University of Michigan, Ann Arbor, MI 48109, USA; 5 Department of Dermatology, Clinic of Orthopaedy, Rheumatology and Dermatology, St. Olavs Hospital, Trondheim University Hospital, Trondheim, Norway; 6 Department of Biostatistics and Center for Statistical Genetics, University of Michigan, Ann Arbor, MI, USA; 7 Department of Computational Medicine and Bioinformatics, Ann Arbor, MI, USA; 8 Center for Statistical Genetics, University of Michigan School of Public Health, Ann Arbor, MI, USA; 9 Analytic and Translational Genetics Unit, Department of Medicine, Massachusetts General Hospital, Boston, MA, USA; 10 Stanley Center for Psychiatric Research, Broad Institute of MIT and Harvard, Cambridge, MA, USA; 11 Department of Epidemiology Research, Statens Serum Institute, Copenhagen, Denmark; 12 Department of Clinical and Molecular Medicine, NTNU Norwegian University of Science and Technology, Trondheim, Norway; 13 BioCore—Bioinformatics Core Facility, NTNU Norwegian University of Science and Technology, Trondheim, Norway; 14 Clinic of Laboratory Medicine, St. Olavs Hospital, Trondheim University Hospital, Trondheim, Norway; 15 Levanger Hospital, Nord-Trøndelag Hospital Trust, Levanger, Norway; 16 Regeneron Genetics Center, Tarrytown, NY 10591, USA; 17 Department of Endocrinology, Clinic of Medicine, St. Olavs Hospital, Trondheim University Hospital, Trondheim 7030, Norway; 18 Department of Human Genetics, University of Michigan, Ann Arbor, MI 48109, USA

**Keywords:** biobank, complex disease, genetics, cardiovascular health, longitudinal, public health, genetic epidemiology, big data, electronic health records, omics

## Abstract

The Trøndelag Health Study (HUNT) is a population-based cohort of ∼229,000 individuals recruited in four waves beginning in 1984 in Trøndelag County, Norway. Approximately 88,000 of these individuals have available genetic data from array genotyping. HUNT participants were recruited during four community-based recruitment waves and provided information on health-related behaviors, self-reported diagnoses, family history of disease, and underwent physical examinations. Linkage via the Norwegian personal identification number integrates digitized health care information from doctor visits and national health registries including death, cancer and prescription registries. Genome-wide association studies of HUNT participants have provided insights into the mechanism of cardiovascular, metabolic, osteoporotic, and liver-related diseases, among others. Unique features of this cohort that facilitate research include nearly 40 years of longitudinal follow-up in a motivated and well-educated population, family data, comprehensive phenotyping, and broad availability of DNA, RNA, urine, fecal, plasma, and serum samples.

## Highlights

• The Trøndelag Health Study (HUNT) is a large, genotyped population-based cohort
• Comprehensive health-related information is available with nearly 40 years follow-up
• A national personal identification number integrates digitized health care information
• GWASs of HUNT participants have provided insights into mechanism of disease

## Graphical abstract

**Figure:**
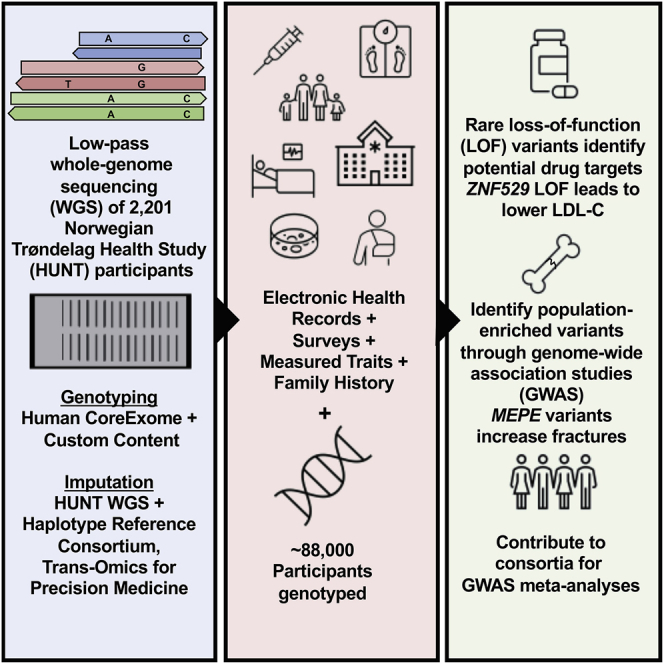

## Teaser

Brumpton et al. present the genetic cohort profile of the Trøndelag Health Study (HUNT), a large, genotyped population-based cohort from Trøndelag County, Norway. They describe the extraordinarily rich features that make it an excellent cohort for genetic research. These include repeated survey data since 1984, broad availability of biological material, and the possibility to link with patient electronic health records and population registries. They show how HUNT has aided in understanding the genetic contribution to human traits and disease and describe future opportunities for research.

## Introduction

**Figure 1:**
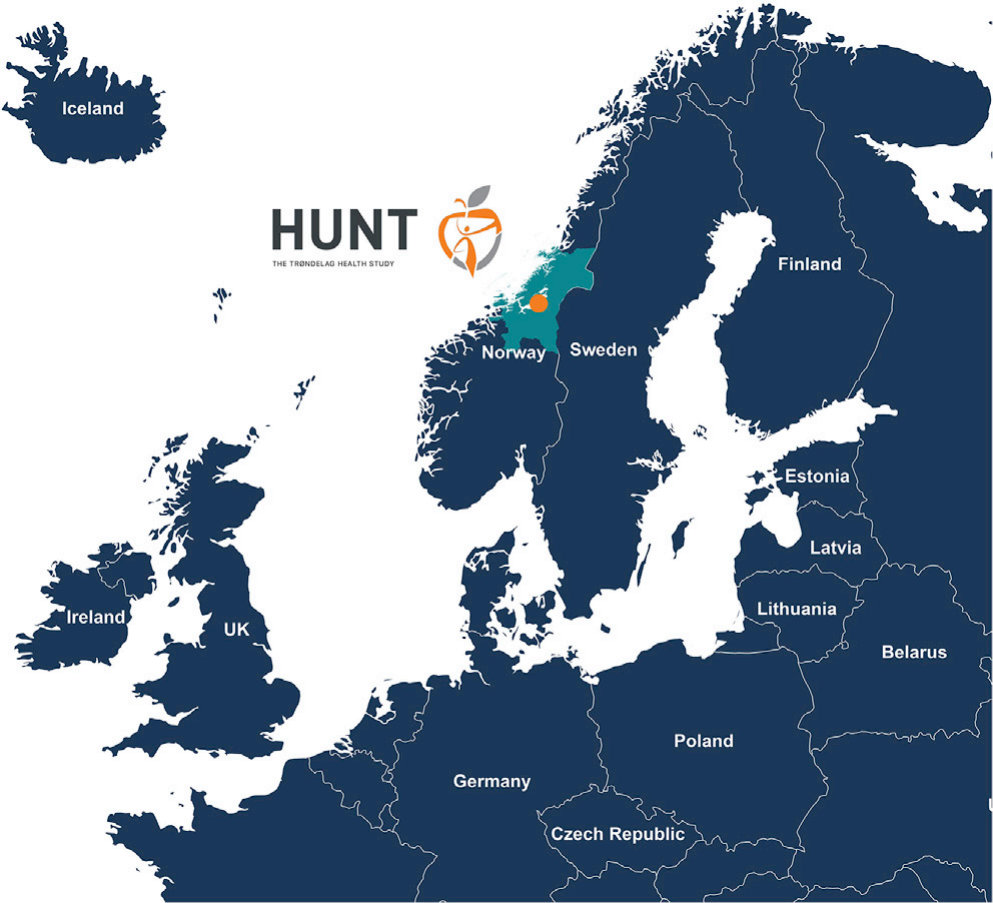
The Trøndelag Health Study (HUNT), Trøndelag, Norway The county of Trøndelag is shaded light blue, and the orange point indicates the location of the HUNT Research Center at Levanger.

Norway, like other Nordic countries, has characteristics that are uniquely favorable for recruitment to population studies, establishing biobanks, and identifying clinical outcomes and disease trajectories. This includes a unique personal identification number applied throughout the life span, a universal and digitized public health care system, and accessible harmonized electronic health records. In addition, 17 mandatory and validated national health registries are used for health analysis, administration, and emergency preparedness, and 52 national medical quality registries provide disease specific data on diagnosis and treatment parameters. Finally, Norwegians are an altruistic, highly motivated population for participating in biomedical research, as reflected in survey response rates of up to 89%. These factors have supported the establishment and maintenance of the Trøndelag Health Study (HUNT), a large population-based prospective Norwegian cohort, linked to registries and biobanks dating back more than 40 years (Figure 1).

To understand the genetic basis of diseases, as well as follow individuals with genetic and epidemiological risk factors in a well-ascertained county in Norway, we established a comprehensive collaboration in 2005 between the HUNT study at the Norwegian University of Science and Technology, Norway, and the University of Michigan, USA (see Data S1). This paper presents the history and status of this collaboration by describing the study population, the strategy incorporating genotyping, sequencing, and imputation-based approaches in HUNT, the vast phenotype data collected by decades of HUNT researchers, the linkage to the digitized public health care system, and key findings to date.

### Study population

**Figure 2:**
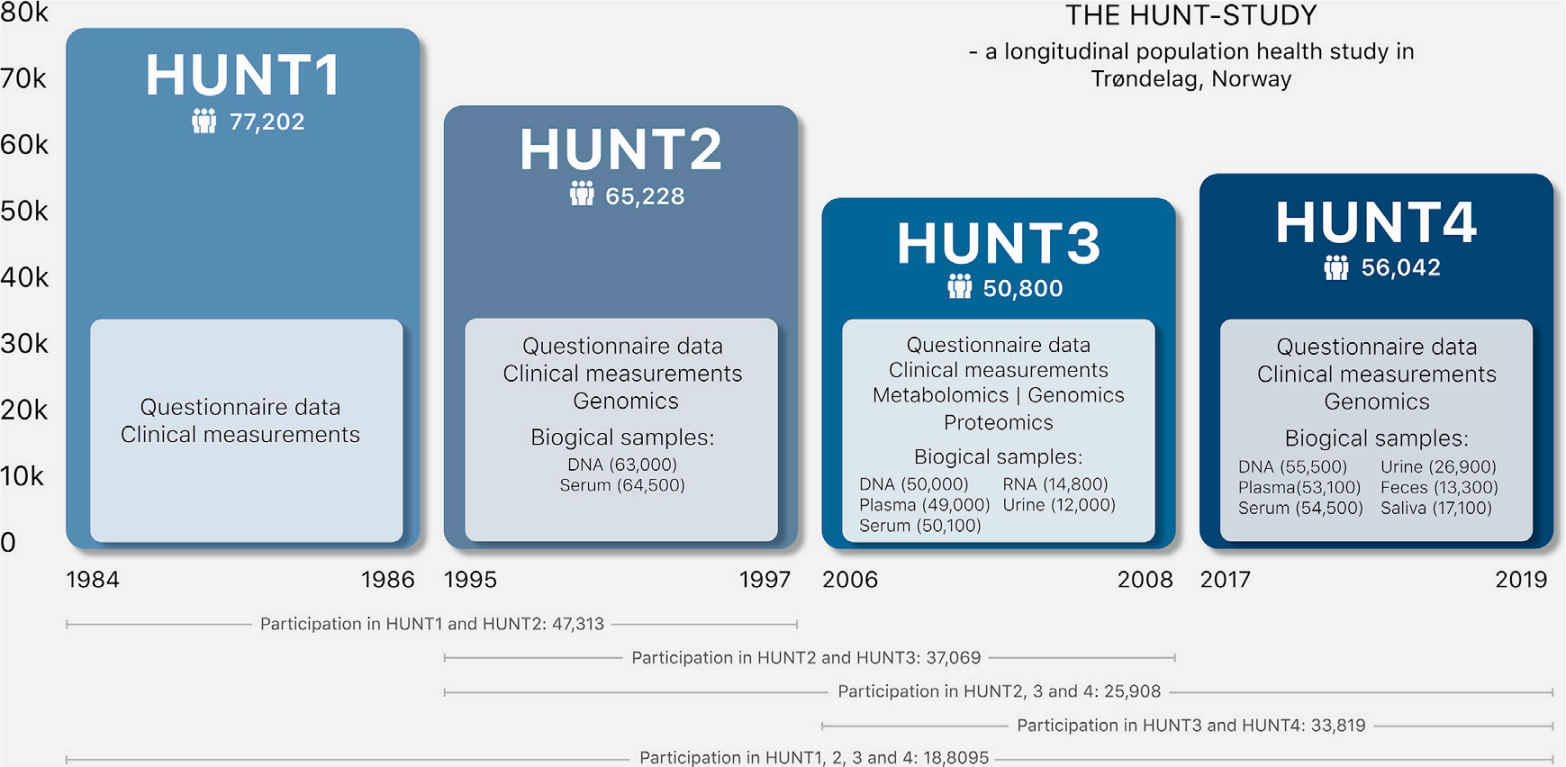
Sample sizes across the HUNT1-4 surveys and details of key data and biological samples DNA, deoxyribonucleic acid; HUNT, Trøndelag Health Study; RNA, ribonucleic acid.

HUNT is an ongoing population-based health study in Trøndelag County, Norway. The study collects health-related data from questionnaires, interviews, and clinical examinations from individuals within this geographical region (Figure 2). More than 229,000 adults (20 years or older at recruitment) have participated in the study to date, of whom 95,000 have provided at least one biological sample (https://www.ntnu.edu/hunt/hunt-samples).^1^, ^2^, ^3^, ^4^ The periodic survey design includes four recruitment waves. HUNT1 (1984–1986), HUNT2 (1995–1997), HUNT3 (2006–2008), and HUNT4 (2017–2019) concentrated primarily on the North-Trøndelag area, where all adults (age ≥ 20 years) were invited. In addition, HUNT4 expanded to collect basic questionnaire data from the adult population of South-Trøndelag (105,797 additional participants).^3^ Approximately 19,000 adults have participated in all four HUNT waves, thus having longitudinal questionnaire and physical exam information spanning over 35 years. Complementing the surveys in adult participants, four separate Young-HUNT surveys gathered data from ∼25,000 adolescents in junior high and high school, concurrent with HUNT2-4. No genotyping has been performed on Young-HUNT; however, 4,212 have sequentially participated in the adult version of HUNT. The HUNT Study has a high level of participation (ranging from 54% to 89% between surveys among those invited) making the cohort a good representative of the general Norwegian population. The HUNT and Young-HUNT cohorts are described in more detail elsewhere.^1^, ^2^, ^3^, ^4^, ^5^

### Genotyping and imputation study design in HUNT

**Figure 3:**
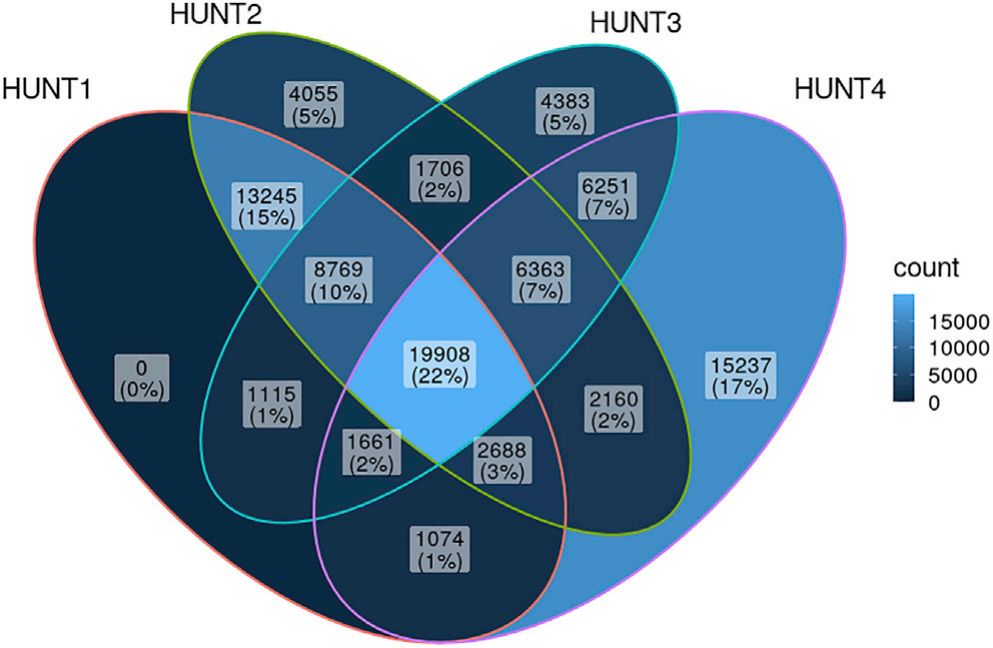
Genotyped samples from HUNT available from the different HUNT surveys (n = 88,615) HUNT, Trøndelag Health Study.

**Figure 4:**
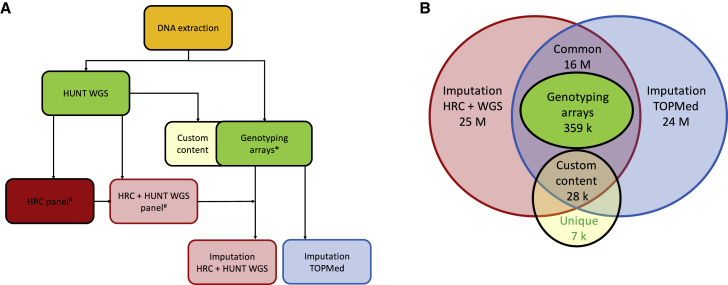
Genotyping and imputation-based approach in HUNT (A) Flowchart of the approach. (B) Number of variants captured by each approach. HUNT, Trøndelag Health Study; HRC, Haplotype Reference Consortium; k, thousand; M, million; TOPMed, Trans-Omics for Precision Medicine; WGS, whole-genome sequencing. ∗Not all genotyping arrays included custom content. ^#^Only 1,200 HUNT samples were sequenced at the time the HRC was established; however, 2,201 were included in the HRC + HUNT WGS panel.

Approximately 88,000 individuals provided DNA for medical research during at least one of the HUNT recruitment periods. Initially, our efforts were focused on identifying genetic variants associated with myocardial infarction (MI).^6^, ^7^, ^8^ Toward this goal, we genotyped exome variants and performed low-pass whole-genome sequencing (4.7× average coverage) in 2014 on 2,201 samples from HUNT2 and HUNT3 (HUNT-WGS) (Table S1), including early-onset MI cases and equal numbers of sex- and age-matched controls. Although no novel significant associations were found, likely due to the limited sample size, this set of low-pass sequences provided important insights into genetic variants present in the Norwegian population and contributed Norwegian reference sequences to the Haplotype Reference Consortium (HRC) imputation panel.^9^ We next completed genome-wide genotyping on all HUNT2-3 participants (n = 70,517) with available DNA (Figure 3). Motivated by a goal of capturing high-quality, common- and low-frequency, and Norwegian-specific variants, we used a variety of approaches to observe or estimate genotypes: (1) direct genotyping using standard and customized HumanCoreExome arrays from Illumina; (2) genotyping and imputation with a merged HRC and HUNT-WGS imputation panel; and (3) imputation with the TOPMed imputation panel (Figure 4). After genotyping 12,864 with standard HumanCoreExome arrays (HumanCoreExome 12 v1.0 and v1.1), we performed genotyping on the remaining samples using a customized HumanCoreExome array (UM HUNT Biobank v1.0), which included protein-altering variants observed in HUNT-WGS. We followed a strict quality control protocol based upon the approach developed by that of Guo et al.^10^ This included excluding samples and variants that failed to reach a 99% call rate, resulting in genotyping 358,964 polymorphic variants. We next used the 2,201 sequenced samples (HUNT-WGS) for joint imputation with the HRC panel.^9^ We previously showed that imputation with a HUNT-specific reference panel improved imputation of low-frequency and population-specific variants compared with using either the 1000 Genomes or HRC reference panels alone.^11^ Finally, we imputed 25 million variants from the TOPMed imputation panel (minor allele count greater than 10), which resulted in slightly lower imputation quality compared with the population-specific reference panel but captured a larger number of variants (Figure S1). These two imputed datasets can be used separately in downstream analysis; we recommend using the HRC and HUNT-WGS imputation for the investigation of the Norwegian-specific variants. Together, the imputations resulted in 33 million variants in 70,517 individuals from HUNT2 or HUNT3, of which 3.3 million variants are not found in UK Biobank. Finally, 18,721 new samples from HUNT4 have recently been genotyped using the same approaches (Human CoreExome array, UM HUNT Biobank v2.0) and following imputation will create a new, larger data freeze of ∼88,000 individuals from HUNT2-4. Further details of the quality control and imputation in HUNT can be found in the STAR Methods.

### Phenotypes

A broad range of phenotypes are available for HUNT participants based on laboratory tests, clinical examinations, and self-reported questionnaires. These include non-fasting blood lipids and glycemic traits; history (including age of diagnosis) of a range of diseases, including cardiovascular events; basic demographics, including sex and participation age; anthropometrics, including weight, height, BMI, and waist-to-hip ratio; blood pressure measurements; and lifestyle information, including smoking status (Table 1). HUNT data categories have been described previously,^2^^,^^3^ and are described in detail on the HUNT databank website (https://www.ntnu.edu/hunt/databank). To ensure data were of high quality, biologic material was handled at the field stations according to appropriate standards and transported to the biobank every evening in a cold chain. Several measurements, including hemoglobin and blood cell counts, creatinine, and cholesterol were sent for immediate analysis, which was performed by specially trained personnel according to the same standardized protocols with the same equipment. Plasma, serum, and buffy coat are stored in aliquots in automated freezers in the HUNT Biobank at −80°C. The databank website describes each measure in more detail, including specific details of the instrument used and coefficients of variation (https://www.ntnu.edu/hunt/databank). Importantly, many measurements and questionnaire items have been intentionally kept identical or similar across HUNT surveys to enable longitudinal analyses, which may contribute to understanding disease progression and survival.

**Table 1:** HUNT cohort demographics for all attendees at HUNT1-4 clinical examinations (N = 123,219), and among those genotyped (N = 88,615)

	All (HUNT1-4)				Genotyped (HUNT2-4)			
	N	Total	Male	Female	N	Total	Male	Female
Number of individuals (%)	123,219		59,121 (48%)	64,098 (52%)	88,615		41,482 (47%)	47,133 (53%)
Age at first attendance, years (range 18–90+; mean ± SD)^a^	123,219	43.8 ± 17.7	43.8 ± 17.3	43.9 ± 18.0	88,566	39.1 ± 14.0	39.2 ± 13.8	39.0 ± 14.1
Age at last attendance, years^b^ (range 18–90+; mean ± SD)	123,219	56.5 ± 19.1	56.1 ± 18.7	56.8 ± 19.5	88,566	55.9 ± 18.6	55.9 ± 18.2	55.8 ± 18.9
Follow-up time, years	123,177	22.4 ± 12.8	22.2 ± 12.9	22.5 ± 12.7	88,548	24.1 ± 12.4	24.2 ± 12.4	24.0 ± 12.5

**Quantitative measurements (mean ± SD)**^b^								

BMI, kg/m^2^	119,888	26.8 ± 4.6	26.9 ± 4.1	26.8 ± 5.1	88,345	27.2 ± 4.7	27.3 ± 4.1	27.0 ± 5.1
SBP^c^, mm Hg	120,448	136.8 ± 23.7	138 ± 21.2	135 ± 25.6	88,420	133 ± 21.3	135 ± 19.2	131 ± 22.8
LDL-C^e^, mg/dL	93,835	3.4 ± 1.1	3.3 ± 1.1	3.4 ± 1.1	87,163	3.3 ± 1.1	3.3 ± 1	3.4 ± 1.1
Creatinine, μmol/L	95,361	80.3 ± 22	88.9 ± 22.7	72.7 ± 18.3	88,527	79.6 ± 22.0	88.3 ± 22.9	72.0 ± 18.0
Glucose^f^, mmol/L	78,429	5.6 ± 1.7	5.7 ± 1.8	5.5 ± 1.6	71,790	5.6 ± 1.7	5.7 ± 1.8	5.5 ± 1.6
Thyroid stimulating hormone, mIU/L	71,213	1.4 ± 1.5	1.5 ± 1.5	1.4 ± 1.6	70,541	1.4 ± 1.5	1.5 ± 1.5	1.4 ± 1.6
Blood hemoglobin, g/dL	54,347	14.6 ± 1.3	15.4 ± 1.2	14.0 ± 1.0	51,892	31 ± 1.7	31.1 ± 1.6	30.8 ± 1.8
FEV1	18,854	3.1 ± 1.0	3.6 ± 1.1	2,7 ± 0.8	17,687	3.1 ± 1.0	3.6 ± 1.1	2.7 ± 0.8
BMD total hip T score HUNT3^g^	11,435	0.1 ± 0.9	–0.1 ± 0.9	0.2 ± 0.9	11,281	0.1 ± 0.9	–0.1 ± 0.9	0.2 ± 0.9

**Socioeconomic status (N,%)**^d^								

Education (%)								
Primary school	39,208	36.1%	17,473 (33.7%)	21,735 (38.3%)	21,235	25.7%	8,801 (22.9%)	12,434 (28.3%)
High School	42,253	38.8%	22,511 (43.3%)	19,742 (34.7%)	36,439	44.2%	19,160 (49.8%)	17,279 (39.3%)
College/University (less than 4 years)	14,765	13.6%	6,819 (13.1%)	7,946 (14.0%)	13,282	16.1%	6,044 (15.7%)	7,238 (16.4%)
College/University (4 or more years	12,565	11.5%	5,161 (9.9%)	7,404 (13.0%)	11,515	14.0%	4,491 (11.6%)	7,024 (16.0%)
Household income, %								
<250,000 NOK	5,620	10.4%	1,777 (7.1%)	3,843 (13.1%)	5,356	10.2%	1,665 (6.9%)	3,691 (12.9%)
250,000 – 450,000 NOK	11,736	21.6%	5,139 (20.6%)	6,597 (22.6%)	11,447	21.7%	4,990 (20.6%)	6,457 (22.6%)
451,000 – 750,000 NOK	15,751	29.1%	7,666 (30.7%)	8,085 (27.6%)	15,362	29.1%	7,453 (30.8%)	7,909 (27.7%)
751,000 – 1,000,000 NOK	11,521	21.3%	5,500 (22.1%)	6,021 (20.6%)	11,260	21.4%	5,360 (22.2%)	5,900 (20.7%)
>1,000,000 NOK	9,564	17.6%	4,863 (19.5%)	4,701 (16.1%)	9,307	17.6%	4,716 (19.5%)	4,591 (16.1%)

FEV1, forced expiratory volume in the first second; HUNT, Trøndelag Health Study; BMI, body mass index; SBP, systolic blood pressure; LDL-C, low-density lipoprotein cholesterol; SD, standard deviation; NOK, Norwegian Krone.

a First attendance of HUNT is reported.

b Last attendance of HUNT is reported.

c Mean of first and second measurements in HUNT1 and mean of second and third measurement in HUNT2, -3, and -4.

d Self-reported education and household income at HUNT.

e Friedewald equation was used to estimate LDL-C.

f Non-fasting glucose.

g ∼70% of participants from HUNT4 also have BMD measured in total hip, which are undergoing quality control.

### Linkage to regional and national health registries

HUNT participants have consented to linkage to the many high-quality health and administrative registries in Norway and to information from medical records. Using the unique personal identification number given to all Norwegian citizens allows for longitudinal follow-up by linkage between HUNT data, regional and national registries, and electronic health records. Norway currently has 17 national health registries (https://helsedata.no/no/) that are mandatory and cover the entire population (Table S2). Commonly used national registries linked with HUNT include the Norwegian Cause of Death Registry (established 1951), the Cancer Registry of Norway (established 1952), the Medical Birth Registry of Norway (established 1967), the Norwegian Prescription Database (established 2004), Norwegian Control and Payment of Health Reimbursements Database (established 2006), and the Norwegian Registry for Primary Health Care (established 2017). Another 52 national disease-specific medical quality registries hold detailed information on treatment and responses at an individual level (https://www.kvalitetsregistre.no/registeroversikt) (Table S2). Electronic health records from the local hospitals hold International Statistical Classification of Diseases and Related Health Problems (ICD) codes back to 1987. Potential linkage to administrative registries expands the data resource, which, among others, includes Statistics Norway, recording income and wealth statistics for individuals and households, and the Norwegian Armed Forces Health Registry (https://helsedata.no). Together, the listed registries provide opportunities to integrate a breadth of data from multiple time points to obtain high-quality phenotypes and related information on, for example, environmental and socioeconomic factors. Time-stamped data allow studies of disease development and progression, such as risk prediction of coronary artery disease.^12^ Some selected disease endpoints are presented in Table 2.

**Table 2:** ICD codes captured in the local hospital register from 1987 to 2021 for selected diseases and the observed case numbers in genotyped HUNT participants

ICD Chapter	ICD-9	ICD-10	Cases genotyped
**Infectious and parasitic diseases**			

COVID-19, virus identified		U07.1	66
Personal history of COVID-19		U08	4
Post COVID-19 condition		U09	11

**Neoplasms**			

Malignant neoplasm of colon, rectosigmoid junction, rectum, anus, and anal canal	153, 154	C18, C19, C20, C21	2,455
Malignant neoplasm of bronchus and lung	162	C34	1,585
Malignant melanoma of skin	172	C43	1,304
Malignant neoplasm of breast	174, 175	C50	2,144
Malignant neoplasm of prostate	185	C61	2,721

**Endocrine, nutritional, and metabolic diseases**			

Hypothyroidism	240, 241, 242, 243, 244, 245	E00, E01, E02, E03	4,269
Type 2 diabetes mellitus	250	E11	7,350

**Mental and behavioral disorders**			

Dementia	290, 294, 331	F00, F01, F02, F03, G30, G31.1	4,431
Mood (affective) disorders	296, 298, 300, 301, 311	F30, F31, F32, F33, F34, F38, F39	9,735

**Diseases of the nervous system**			

Parkinson disease	332, 333	G20, G21, G22, F02.3	1,177
Epilepsy	345	G40	2,296
Migraine	346	G43	2,758

**Diseases of the eye and adnexa**			

Glaucoma	365	H40	5,610

**Diseases of the circulatory system**			

Essential (primary) hypertension	401	I10	20,031
Angina pectoris	413.9	I20	7,660
Acute myocardial infarction	410	I21	7,005
Atrial fibrillation and flutter	427	I48	10,232
Heart failure	428	I50, I09, I11	7,117
Intracerebral hemorrhage	431, 432	I61	1,082
Aortic aneurysm and dissection	441	I71	2,090

**Diseases of the respiratory system**			

Chronic obstructive pulmonary disease	496	J44.8, J44.9	5,188
Asthma	493	J45	5,636
Post-inflammatory pulmonary fibrosis	515	J84.1, J84.8	296

**Diseases of the digestive system**			

Crohn disease	555	K50	772
Ulcerative colitis	556	K51	1,971
Celiac disease	579	K90.0	1,088

**Diseases of the skin and subcutaneous tissue**			

Atopic dermatitis	691.8	L20	1,134
Psoriasis	696	L40	2,782

**Diseases of the musculoskeletal system and connective tissue**			

Gout	274	M10	1,694
Ankylosing spondylitis	720	M45	1,666

**Diseases of the genitourinary system**			

Chronic kidney disease	585	N18	3,464

**Pregnancy, childbirth and the puerperium**			

Gestational hypertension	342	O13	609

**Prescription data**^a^			

Low dose aspirin	–	–	22,500
Statin	–	–	2,200

Numbers are from a data query (August 8, 2021) from the Nord-Trøndelag Hospital Trust, including St. Olavs, Namsos, and Levanger Hospitals of participants selected for genotyping from HUNT2-4. The register is ongoing and therefore the number of cases continues to increase over time. Numbers based on hospital data are likely to result in under-ascertainment of less-serious common conditions. HUNT, Trøndelag Health Study; ICD, International Statistical Classification of Diseases and Related Health Problems.

a Approximate numbers from the prescription register and restricted to those genotyped in HUNT2-3 only.

### Analytical approaches with related samples

The majority of HUNT participants are of Norwegian ancestry.^4^ Using principal components of ancestry projected onto the Human Genome Diversity Project, we typically exclude samples of non-European ancestry (<2%) (Figure S2) due to limited power. We have observed fine-scale differences between North- and South-Trøndelag and between individuals born closer to the coast versus the border with Sweden.^13^ In addition, because of high ascertainment from a single county in Norway (Trøndelag), there are many related individuals within the cohort. A total of 79,551 (89%) out of 88,615 HUNT2-4 participants have at least one second-degree or closer relative who also participates in HUNT (Figure S3; Table S3). High degree of participant relatedness in the dataset on one hand allows for unique data analysis methods using nuclear or extended families but can result in bias when using methods that assume unrelated individuals or power loss if related individuals are excluded. An early effort to use extended families and genetic data in HUNT was for the analysis of rare coding variants,^14^ where family samples can provide more power to detect associations when sample sizes were limited and only a modest fraction of all trait-associated variants were identified.^14^

Previously, methods had been developed to account for relatedness for analysis of quantitative traits,^15^ but methods to properly account for relatedness and control for unbalanced case-control ratios for binary traits were lacking. We therefore developed statistical methods to allow for the analysis of all individuals, and to control for case-control imbalance of binary phenotypes, which is commonly observed in biobanks, such as HUNT. These methods, which are computationally efficient in biobank-scale data, allowed us to perform association testing in HUNT for both single variants (using SAIGE) and gene-based burden tests (using SAIGE-GENE) while accounting for sample relatedness with a sparse identical by state sharing matrix.^14^^,^^16^, ^17^, ^18^ These methods account for case-control imbalance of binary phenotypes, typical in a population-based sample, by using the saddlepoint approximation to calibrate unbalanced case-control ratios in score tests based on logistic mixed models.^14^ We demonstrated a vast improvement in reducing type I error rates when analyzing unbalanced case-control ratios with SAIGE in HUNT. For example, venous thromboembolism, with 2,325 cases and 65,294 controls and a case-control ratio of 0.036 had substantial inflation of type I error with methods available prior to the development of SAIGE (Figure S4). To demonstrate the application of SAIGE-GENE, we investigated 13,416 genes, with at least 2 rare (MAF ≤ 1%) missense and/or stop-gain variants that were directly genotyped or imputed from the joint HRC and HUNT-WGS reference panel among 69,716 Norwegian samples from HUNT2-3 with measured high-density lipoprotein. We identified eight genes with p values below the exome-wide significance threshold (p ≤ 2.5 × 10^−6^), seven of which remained significant after conditioning on nearby single-variant associations, suggesting independent rare coding variants within these genes.^17^ Importantly, using SAIGE and SAIGE-GENE, we were able to use all samples, account for sample relatedness case-control imbalance, and maintain well-controlled type I error rates.

A traditional way of using related samples is linkage analysis, which, however, has computational challenges in the era of whole-genome genetics. To allow for linkage testing in datasets with millions of genetic markers, faster and computationally scalable linkage analysis methods have been developed, e.g., Population Linkage.^19^ Population Linkage uses a Haseman-Elston regression (originally used for sibling pair linkage analysis) to estimate variance components from pairwise relationships and identity by descent estimates. Using HUNT data, Zajac et al. observed 25 significant linkage peaks with LOD > 3 across 19 distinct loci for the four traits (high-density lipoprotein, low-density lipoprotein, total cholesterol, and triglycerides), where 5 peaks with LOD > 3 were not replicated at genome-wide significance in a genome-wide association study (GWAS) of 359,432 genotyped variants in HUNT.^19^ However, after imputing the dataset with the HRC and HUNT-WGS reference panel to cover more variants or meta-analysis in the Global Lipids Genetics Consortium, significant associations in all five linkage peaks were observed. This study demonstrates one of the benefits of linkage analysis over GWAS, which is the ability to test for linkage in regions that are difficult to genotype, such as rare variants, structural variants, copy number variants, or variants in highly repetitive regions, as long as identical-by-descent segments in the region can be identified.^19^ Finally, linkage analysis may improve statistical power when investigating rare risk variants that segregate within families and reduce confounding effects of population stratification.

The high degree of relatedness in the HUNT Study participants has enabled analysis methods tailored to this study design. These include GWAS by proxy,^20^^,^^21^ in which the phenotypes of non-genotyped family members of genotyped HUNT participants can be used to identify proxy-cases, individuals with a proportion (0.5 for first-degree relatives) of the genetic risk of cases. These proxy-cases can be appropriately modeled to increase the statistical power in GWAS. For example, the power to detect an allele with an odds ratio of 1.1 and MAF of 0.21 at an alpha of 5 × 10^−8^ increases from 0.419 to 0.644 when proxy-cases were appropriately modeled instead of used as controls in standard GWAS (Figure S5A). We also present empirical results for a known type 2 diabetes variant rs7903146 in TCF7L2 in HUNT (Figure S5B).

## Results

### Genetic discoveries from HUNT

The wealth of phenotypic and genetic data available in the HUNT cohort has led to the discovery of many new genetic associations across a broad range of traits (Table 3). Early genetic studies of HUNT participants used exome arrays and focused on cardiovascular disease. We identified a novel coding variant in *TM6SF2* associated with total cholesterol, MI, and liver enzymes^6^ and replicated known MI associations at the 9p21 locus and a low-frequency missense variant in the *LPA* gene (p.Ile1891Met).^7^ Following the genotyping of nearly 70,000 participants in HUNT2 and HUNT3 and the development of a combined HRC and HUNT-WGS imputation reference panel, we extended our analyses to a genome-wide search. Through imputation of indels called from low-pass HUNT-WGS, we discovered a rare mutation in the *MEPE* gene, enriched in the Norwegian population (0.8% in HUNT, 0.1% in non-Finnish Europeans), that was associated with low forearm bone mineral density and increased risk of osteoporosis and fractures.^22^ Although this region had been identified previously as associated with bone mineral density,^23^ the association in HUNT with replication in the UK Biobank^24^ pin-pointed *MEPE* as the likely causal gene in the region by identifying an insertion/deletion polymorphism that likely resulted in a loss-of-function protein. In another study, we paid special attention to loss-of-function mutations associated with favorable blood lipid profiles (reduced LDL cholesterol and reduced CAD risk), which were not associated with altered liver enzymes or liver damage. We also found an elderly individual with homozygous *ZNF529* loss-of-function variant showing no signs of cardiovascular disease or diabetes, suggesting that the full knockout of this gene is viable. This highlighted *ZNF529* as a potential therapeutic target for lipids^25^ identified from sequencing and custom content genotyping.

**Table 3:** Genetic discoveries across HUNT genotyping and analysis strategies

Strategy	Frequency range/number of variants	Benefits	Exemplary papers
Genotyping with custom exome content designed from HUNT sequenced samples (UM HUNT Biobank Array)	rare–common/80,137–358,964	identify low-frequency variants not amenable to imputation-based approaches	identified likely causal gene, *TM6SF2*, associated with TC and MI^6^
			found LOF variant in *ZNF529* that leads to lower LDL-C^25^
HRC and HUNT-WGS Imputation from Human CoreExome Array	low–common/22 million	include population-specific variants through improved imputation	population-enriched variant in *MEPE* pinpoints causal gene for fracture risk^22^
			identified variants associated with thyroid function,^26^ kidney function,^27^ serum PCSK9,^28^ and atrial fibrillation^29^^,^^30^
TOPMed Imputation from Human CoreExome Array	low–common/25 million	expand number of available variants for association testing	identified variants associated with troponin and serum iron in the general population^31^^,^^32^
Family-based design, >15,000 sibling pairs, >35,000 parent-offspring	any/10–1,000	improve effect size estimates and test traits among un-studied relatives	introduced new analysis methods, including SAIGE,^16^ within-family Mendelian randomization, and GWAS^33^^,^^34^
Mendelian randomization	any/10–1,000	identify causal links between environmental factors (genetically determined traits) and outcomes	explored the role of lipids and apolipoproteins on kidney function^35^^,^^36^
			demonstrated an inverse association between thyroid-stimulating hormone and thyroid cancer^26^

Note: rare variants <1% minor allele frequency (MAF); low-frequency variants, 1%–5% MAF; common variants, >5% MAF. HUNT, Trøndelag Health Study; HRC, Haplotype Reference Consortium; LDL-C, low density lipoprotein cholesterol; LOF, loss of function; MI, myocardial infraction; TC, total cholesterol; TOPMed, Trans-Omics for Precision Medicine; UM, University of Michigan; WGS, whole-genome sequencing.

On top of the association studies performed using HUNT data only, we have contributed to many international consortium efforts aimed at aggregating GWAS data across cohorts. By performing GWAS meta-analyses that included HUNT and other cohorts, efforts driven by our research team have identified genetic variants associated with atrial fibrillation that may act through a mechanism of impaired muscle cell differentiation and tissue formation during fetal heart development^29^ and cardiac structural remodeling^30^; variants associated with estimated glomerular filtration rate exhibiting a sex-specific effect^27^^,^^37^; and variants associated with thyroid-stimulating hormone that revealed an inverse relationship between TSH levels and thyroid cancer.^26^ Later studies using the TOPMed reference panel^38^ identified variants associated with circulating cardiac troponin I level, investigated its role as a non-causal biomarker for MI using Mendelian randomization,^31^ and identified variants associated with iron-related biomarker levels and explored their relationship with all-cause mortality.^32^

### Causal inference and family effects

The high degree of relatedness in the HUNT Study offers a unique opportunity to use family-based designs to investigate causal associations. Mendelian randomization (MR), which uses genetic variants as instrumental variables to investigate modifiable (non-genetic) factors, was first proposed using parent-offspring designs.^39^ Alleles that are inherited from each parent are randomly determined during the meiotic process. This random allocation is essential to providing reliable comparisons in MR studies. However, due to the lack of genotyped family data, previous studies applied MR on the population-level, where the random allocation of alleles is only approximate. We were able to use the ∼15,000 families in HUNT to perform MR as originally proposed—in family-based designs.^33^ Using this approach in HUNT, we showed empirically that MR estimates from samples of unrelated individuals for the association of taller height and lower BMI increase educational attainment, were likely induced by population structure, assortative mating, or dynastic effects. We observed no clear associations in within-family MR analyses in HUNT or in a replication cohort of 222,368 siblings from 23andMe.^33^ This approach has since grown in popularity and, together with HUNT, many cohorts now contribute to the investigation of causal associations with family-based designs.^34^

Further leveraging the family structure information in HUNT, we have performed and have future opportunities to investigate causal effects between family members, for example parent-offspring effects^40^^,^^41^ and assortative mating and sibling effects.^42^ These study designs have not been previously possible due to the lack of genotyped family data, and this has limited both causal inference (as mentioned above) and the ability of typical GWASs to distinguish between direct and indirect genetic effects.^34^ HUNT data allow for study designs to disentangle these sources of genotype-phenotype associations in humans. In one such example, we used 26,057 mother-offspring and 9,792 father-offspring pairs to investigate whether adverse environmental factors *in utero* increased future risk of cardiometabolic disease in the offspring. We observed that adverse maternal intrauterine environment, as proxied by maternal SNPs that influence offspring birthweight, were unlikely to be a major determinant of late-life cardiometabolic outcomes of the offspring.^40^

### Contribution to collaborative studies

While the HUNT study has been an essential cohort in the genetic discoveries and causal inference mentioned so far, used in isolation it is limited due to low power to investigate uncommon phenotypes, uncertainty of the generalizability of findings to non-Europeans, and the lack of an independent sample for replication. To overcome these limitations, we contribute to genetic studies worldwide through participation in consortia focused on a variety of diseases including cardiovascular disease,^43^^,^^44^ lipids,^45^^,^^46^ type 2 diabetes,^47^ osteoporosis,^48^ decline in kidney function,^49^ Alzheimer’s disease,^50^ bipolar disease,^51^ intracranial aneurysms,^52^ insomnia,^53^ respiratory health,^54^ and sleepiness.^55^ We also contributed HUNT data to studies of anthropometric traits,^56^ alcohol and nicotine use,^57^^,^^58^ COVID-19,^59^ phenome-wide discovery,^60^ and genetic risk prediction,^12^ among others. These contributions highlight efforts from researchers in equal parts from the K.G. Jebsen Center for Genetic Epidemiology, NTNU, Norway, the University of Michigan Medical School, and the University of Michigan School of Public Health, USA. We believe that team science by consortia^60^ fulfills the goals of the HUNT study and moves the science fastest toward new discoveries and improved human health.

## Discussion

### Limitations of the study

As noted above, the HUNT study includes primarily individuals of European descent and lacks diverse ancestries for study. In addition, it is limited by sample size to investigate uncommon phenotypes. Furthermore, while all residents aged ≥20 were invited to attend HUNT, biological samples were not available for all participants, which may limit generalizability. However, the relatively high level of participation in HUNT, compared with other studies, indicates a lower concern for selection bias.

### Summary

Together, the multifaceted genetic discovery strategy incorporating genotyping, sequencing, and imputation-based approaches in HUNT has aided the identification of likely causal genes and variants for disease and human traits. It has also proved to be a valuable resource for genetically informed methods of causal inference, supporting the identification of modifiable risk factors. We owe this success to the willingness and high participation rates of the people of Trøndelag, the vast phenotyping collected by decades of HUNT researchers, and access to digitized public health care systems. We hope that initiatives such as this, which capture population-specific variants, use up to 40 years of existing longitudinal biomedical research data, and where the majority of adult inhabitants participated, make a strong case for why it is important to have genetic data both in Norway and a wide range of populations. We anticipate that the rich data collection will continue to be a unique dataset for future opportunities in longitudinal and family-based designs, genetic discoveries, Mendelian randomization, meta-analysis and polygenic score validation, well into the future.

## STAR★Methods

### Key resources table

**Table:** 

REAGENT or RESOURCE	SOURCE	IDENTIFIER
**Critical commercial assays**		

HumanCoreExome12 v1.0	Illumina	https://support.illumina.com/downloads/humancoreexome-12v1-0_product_files.html
HumanCoreExome12 v1.1	Illumina	https://support.illumina.com/downloads/humancoreexome-12-v1-1-product-files.html
UM HUNT Biobank v1.0	Illumina	https://support.illumina.com/downloads/humancoreexome-24-v1-0-product-files.html Note: The exact base array HumanCoreExome-24 v1.1 is not online
UM HUNT Biobank v2.0	Illumina	https://support.illumina.com/downloads/infinium-coreexome-24-v1-3-product-files.html

**Deposited data**		

Cambridge Reference Sequence of the human mtDNA	The ENCODE Project Consortium^61^	http://genome.ucsc.edu
Genome Reference Consortium Human genome build 37	The ENCODE Project Consortium^61^	http://genome.ucsc.edu
Haplotype Reference Consortium Release 1.1	McCarthy et al.^9^	https://ega-archive.org/datasets/EGAD00001002729
Post processed Human Genome Diversity Project data	Wang et al.^62^	http://csg.sph.umich.edu/chaolong/LASER
The Trøndelag Health Study (HUNT) genetic data	This paper	https://www.ntnu.edu/hunt
The Trøndelag Health Study (HUNT). The HUNT survey data may be accessed by application to the HUNT Research Center	Åsvold et al.^3^	https://www.ntnu.edu/hunt
The Trøndelag Health Study (HUNT) summary statistics	This paper	https://dataverse.no
The Trøndelag Health Study (HUNT) Willer Lab summary statistics	This paper	https://csg.sph.umich.edu/willer/public
TOPMed	Taliun et al.^38^	See Extended Data Table 2 for dbGaP study phs IDs

**Software and algorithms**		

BAF Regress	Jun et al.^63^	https://genome.sph.umich.edu/wiki/BAFRegress
BLAT	Kent^64^	http://genome.ucsc.edu; RRID:SCR_011919
Eagle2 v2.3	Loh et al.^65^	https://www.hsph.harvard.edu/alkes-price/software/
FRAPOSA	Zhang et al.^66^	https://github.com/daviddaiweizhang/fraposa
GenomeStudio	Illumina	https://support.illumina.com/array/array_software/genomestudio/downloads.html, RRID;SCR_010973
Minimac3	Das et al.^73^	https://genome.sph.umich.edu/wiki/Minimac3; RRID:SCR_009292
Minimac4	Das et al.^73^	https://genome.sph.umich.edu/wiki/Minimac4; RRID:SCR_009292
PLINK v1.90	PLINK Working Group^67^	https://www.cog-genomics.org/plink/1.9; RRID:SCR_001757
SAIGE	Zhou et al.^16^	https://github.com/weizhouUMICH/SAIGE
SAIGE-GENE	Zhou et al.^17^	https://github.com/weizhouUMICH/SAIGE

### Resource availability

#### Lead contact

Further information should be directed to and will be fulfilled by the lead contact, Ben Brumpton (ben.brumpton@ntnu.no).

#### Materials availability

This study did not generate new unique reagents or material.

### Experimental model and subject details

All residents in North-Trøndelag area (age ≥20 years), were invited to HUNT1-4. In addition, HUNT4 expanded to collect basic questionnaire data from the adult population of South-Trøndelag as described elsewhere.^3^ Sample size, sex, gender, and information about age for HUNT1-4 are provided for study participants in Table 1.

The genotyping in HUNT and work presented in this cohort profile was approved by the Regional Committee for Ethics in Medical Research, Central Norway (2014/144, 2018/1622, 152,023). All participants signed informed consent for participation and the use of data in research.

### Method details

#### Genotyping array design

We aimed to identify as many high-quality genetic variants among HUNT participants as possible. Toward this aim, we developed a list of custom content for inclusion on one of four Illumina Human Core Exome arrays (HumanCoreExome12 v1.0, HumanCoreExome12 v1.1, UM HUNT Biobank v1.0 and UM HUNT Biobank v2.0) to directly genotype (i) 16,116 missense and loss-of-function variants as well as 1,072 lipid-associated variants identified from low-pass sequencing, (ii) 149 variants observed in Norwegian clinics for familial hypercholesterolemia, (iii) 5,324 Neanderthal variants, and (iv) 32,868 not-previously-observed variants predicted to introduce a premature stop codon in any of 56 genes in which protein-altering variants are deemed clinically actionable by The American College of Medical Genetics and Genomics (ACMG56)^68^ (Table S4). Additionally, for the genotyping of HUNT4, we included variants for traits of interest including psoriasis, depression, alcohol use disorder, breast cancer, liver function, and bone mineral density; variants in the GWAS catalog; and loss-of-function variants available in TOPMed but poorly imputable in HUNT samples.

#### Genotyping procedures

Protocols were carefully planned to mitigate any possible batch effects from the genotyping process. Sample assignments to plates and plate positions were randomized and sample sets that needed to be grouped together (e.g., based on robot requirements for liquid volume handling or a requirement for re-precipitation of DNA, etc.) were randomized within each subgroup. Within each plate, genetically determined sex was evaluated against expected sex to identify any plate orientation issues. To enable this during genotype calling, new HUNT-specific cluster files were developed for the genotyping arrays using GenomeStudio, which had to be specific to each array version. Following genotype calling, allele frequencies were examined between array versions and any variants that demonstrated significant association with batch or array versions were excluded. Limited manual validation of GenomeStudio calls (a few thousand variants) were performed. Quality control was performed based upon the approach developed by that of Guo et al.^10^

After a first round of automatic clustering in GenomeStudio (including samples with call rate >95%), samples that failed to reach a 99% call rate, had contamination >2.5% as estimated with BAF Regress,^63^ large chromosomal copy number variants, lower call rate of a technical duplicate pair and twins, gonosomal constellations other than XX and XY, or whose inferred sex contradicted the reported gender, were excluded. Samples that passed quality control were analyzed in a further round of genotype calling following the Genome Studio quality control protocol described elsewhere.^10^ Genomic position, strand orientation and the reference allele of genotyped variants were determined by aligning their probe sequences against the human genome (Genome Reference Consortium Human genome build 37 and revised Cambridge Reference Sequence of the human mtDNA; http://genome.ucsc.edu) using BLAT.^61^ Variants were excluded if (1) their probe sequences could not be perfectly mapped to the reference genome, cluster separation was <0.3, Gentrain score was <0.15, showed deviations from Hardy Weinberg equilibrium in unrelated samples of European ancestry with p value < 0.0001), their call rate was <99%, or another assay with higher call rate genotyped the same variant. Ancestry of all samples was inferred by projecting all genotyped samples into the space of the principal components of the Human Genome Diversity Project (HGDP) reference panel (938 unrelated individuals; downloaded from http://csg.sph.umich.edu/chaolong/LASER/).^62^^,^^64^ For genotyping batches from HUNT2 and HUNT3, PLINK v1.90^69^ was used and recent European ancestry was defined as samples that fell into an ellipsoid spanning exclusively European populations of the HGDP panel. For genotyping from HUNT4, we predicted ancestry using an online singular value decomposition and shrinkage adjustment algorithm (FRAPOSA) with the same reference panel.^67^ The different arrays were harmonized by reducing to a set of overlapping variants and excluding variants that showed frequency differences >15% between datasets, or that were monomorphic in one and had MAF >1% in another dataset. The resulting genotype data were phased using Eagle2 v2.3^71^.

#### Imputation

The imputation described here is limited to the 69,716 samples of recent European ancestry from HUNT2-3, as the work on HUNT4 is ongoing. Samples were imputed using Minimac3 (v2.0.1, http://genome.sph.umich.edu/wiki/Minimac3)^65^ with default settings (2.5 Mb reference-based chunking with 500kb windows) and the HUNT-WGS customized Haplo-type Reference consortium release 1.1 (HRC v1.1) for autosomal variants and HRC v1.1 for chromosome X variants.^9^ The HUNT-WGS customized reference panel represented the merged panel of two reciprocally imputed reference panels: (1) 2,201 low-coverage (5x) whole-genome sequenced samples from the HUNT study (HUNT-WGS) and (2) HRC v1.1 with 1,023 overlapping HUNT WGS samples removed before merging. Since only 1,200 HUNT samples were sequenced at the time the HRC was established, we instead merged all HUNT-WGS samples (including indels) with the non-HUNT HRC samples to create a combined HRC and HUNT-WGAS imputation reference panel. Additionally, we recently performed imputation from 60,039 TOPMed reference genomes using Minimac4 (v1.0).

## Data Availability

The HUNT data reported in this study cannot be deposited in a public repository because it is governed by Norwegian law. To request access, researchers associated with Norwegian research institutes can apply for the use of HUNT data and samples with approval by the Regional Committee for Medical and Health Research Ethics. Researchers from other countries may apply if collaborating with a Norwegian Principal Investigator. Information for data access can be found at https://www.ntnu.edu/hunt/data. The HUNT variables are available for browsing on the HUNT databank at https://hunt-db.medisin.ntnu.no/hunt-db/. Use of the full genetic dataset requires the use of an approved secure computing solution such as the HUNT Cloud (https://docs.hdc.ntnu.no). Data linkages between HUNT and health or administrative registries require that the principal investigator has obtained project-specific approval for such linkage from the Regional Committee for Medical and Health Research Ethics, Norway and each registry owner. Summary statistics derived from HUNT have been deposited at (DataverseNO: https://dataverse.no/) and the Willer lab (Willer lab: http://csg.sph.umich.edu/willer/public/) and are publicly available as of the date of publication. This paper does not report original code. DOIs for pre-existing code used in this paper is listed in the Key resources table. Any additional information required to reanalyze the data reported in this paper is available from the lead contact upon request.

## Supplemental information

Document S1. Figures S1–S5, Tables S1, S3, and S4, and Data S1

Table S2. Overview of mandatory national registries, other national and regional registries, related to Table 2

Document S2. Transparent peer review records for Brumpton et al

Document S3. Article plus supplemental information
