# Supplementary material for: The HUNT study: A population-based cohort for genetic research
Source: Cell Genom. 2022 Oct 12;2(10):100193. doi: 10.1016/j.xgen.2022.100193 (PMC9903730; doi:10.1016/j.xgen.2022.100193)
Supplement: Document S1. Figures S1–S5, Tables S1, S3, and S4, and Data S1 [file mmc1.pdf]

Cell Genomics, Volume *2*

Supplemental information

The HUNT study: A population-based cohort for genetic research

Ben M. Brumpton, Sarah Graham, Ida Surakka, Anne Heidi Skogholt, Mari Løset, Lars G. Fritsche, Brooke Wolford, Wei Zhou, Jonas Bille Nielsen, Oddgeir L. Holmen, Maiken E. Gabrielsen, Laurent Thomas, Laxmi Bhatta, Humaira Rasheed, He Zhang, Hyun Min Kang, Whitney Hornsby, Marta Riise Moksnes, Eivind Coward, Mads Melbye, Guro F. Giskeødegård, Jørn Fenstad, Steinar Krokstad, Marit Næss, Arnulf Langhammer, Michael Boehnke, Gonçalo R. Abecasis, Bjørn Olav Åsvold, Kristian Hveem, and Cristen J. Willer

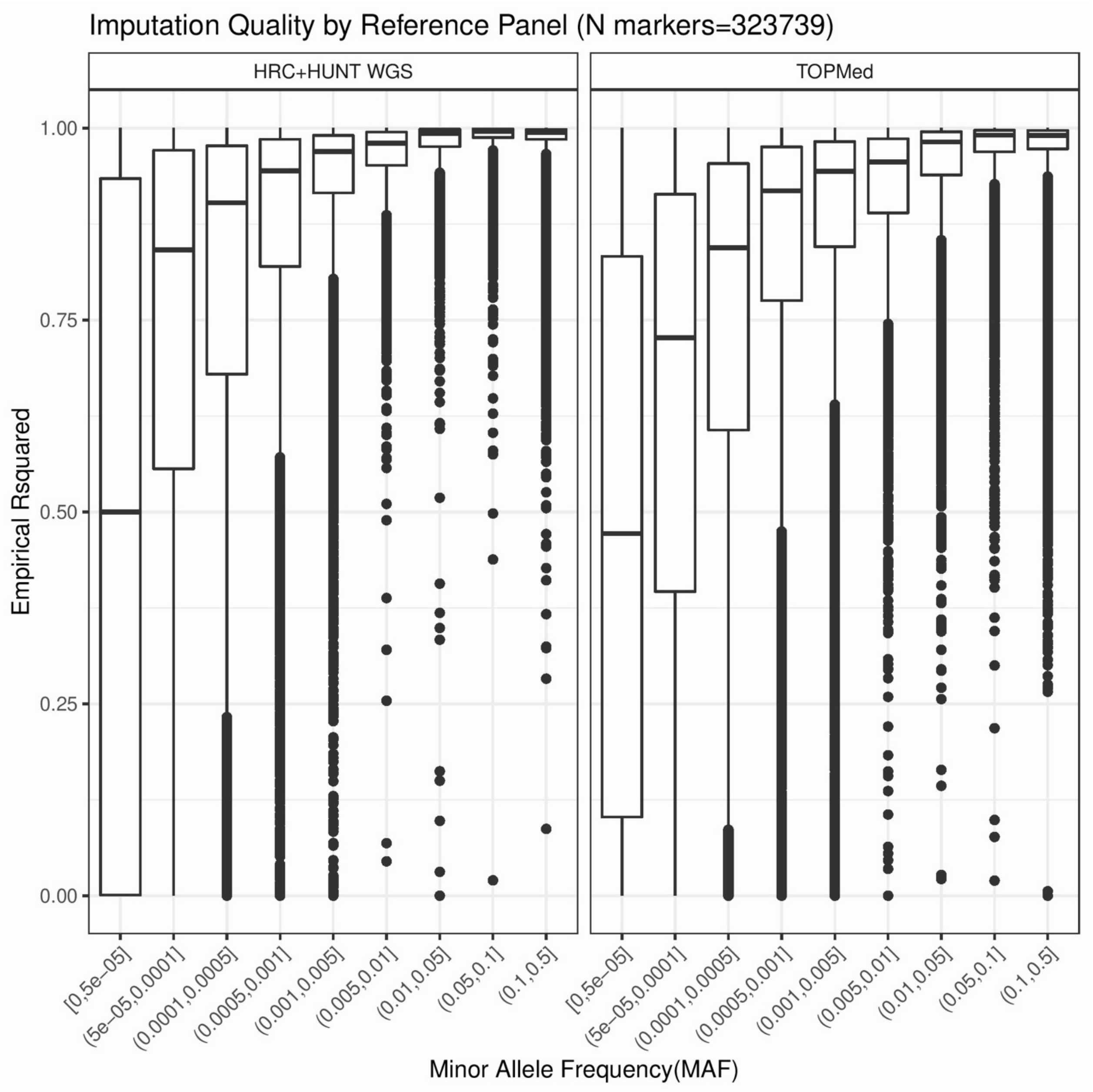

Figure S1. Imputation quality from HRC+HUNT WGS and TOPMed imputation panels in HUNT2-3. Related to STAR Methods (Imputation).

HRC: Haplotype Reference Consortium, HUNT: Trøndelag Health Study, TOPMed: Trans-Omics for Precision Medicine, WGS: Whole genome sequencing

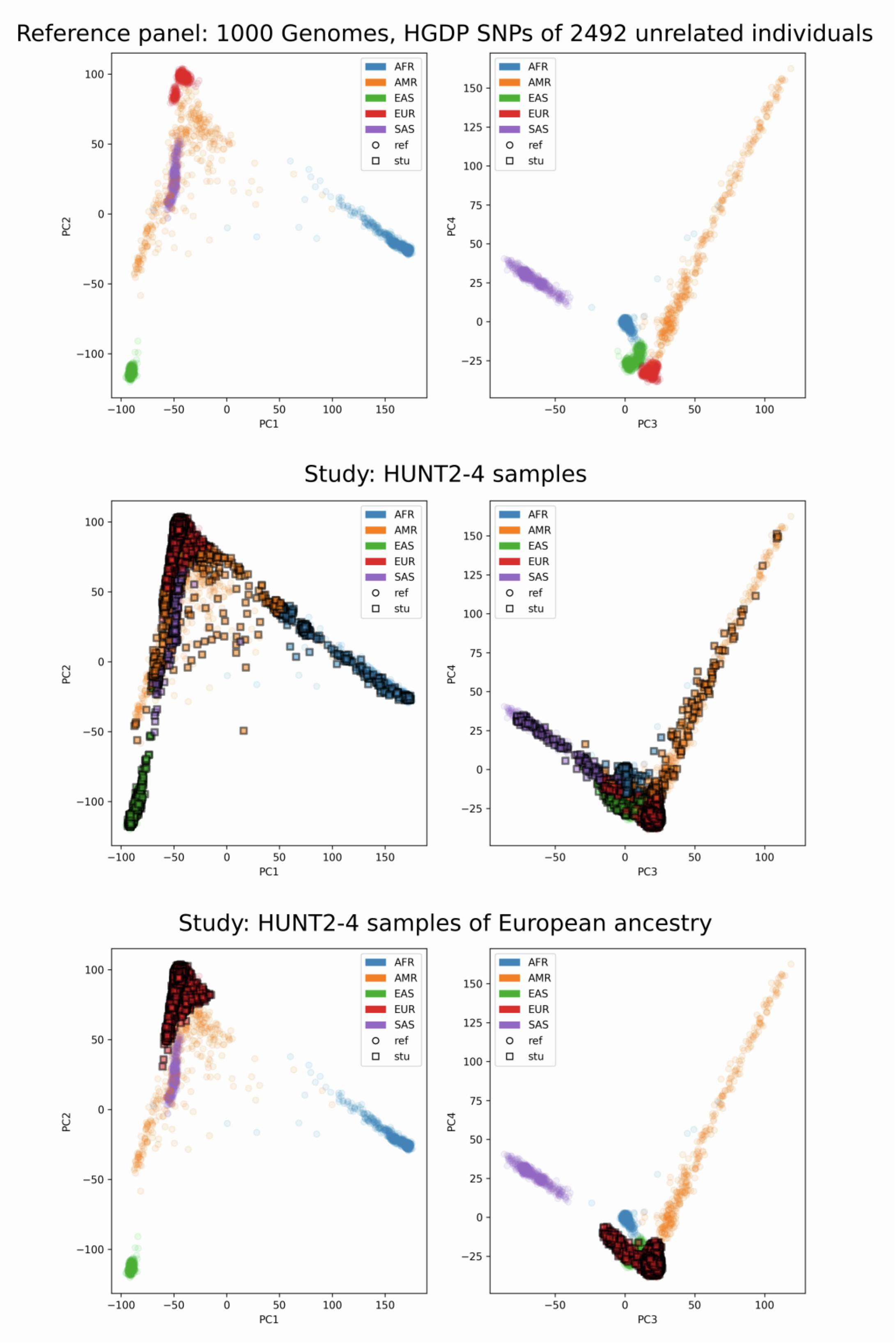
Reference panel: 1000 Genomes, HGDP SNPs of 2492 unrelated individuals
Study: HUNT2-4 samples
Study: HUNT2-4 samples of European ancestry
AFR
AMR
EAS
EUR
SAS
ref
stu
PC1
PC2
PC3
PC4

Figure S2. Plot of the first 4 principal components of ancestry projected with FRAPOSA onto the 1000 Genomes Project for the genotyped HUNT2-4 samples (N=88 615). 1,571 (<2%) samples with non-European ancestries were excluded from further genetic studies. Related to STAR Methods (Genotyping Procedures).

HGDP: Human Genome Diversity Project, HUNT: Trøndelag Health Study, SNP: Single-nucleotide polymorphism.

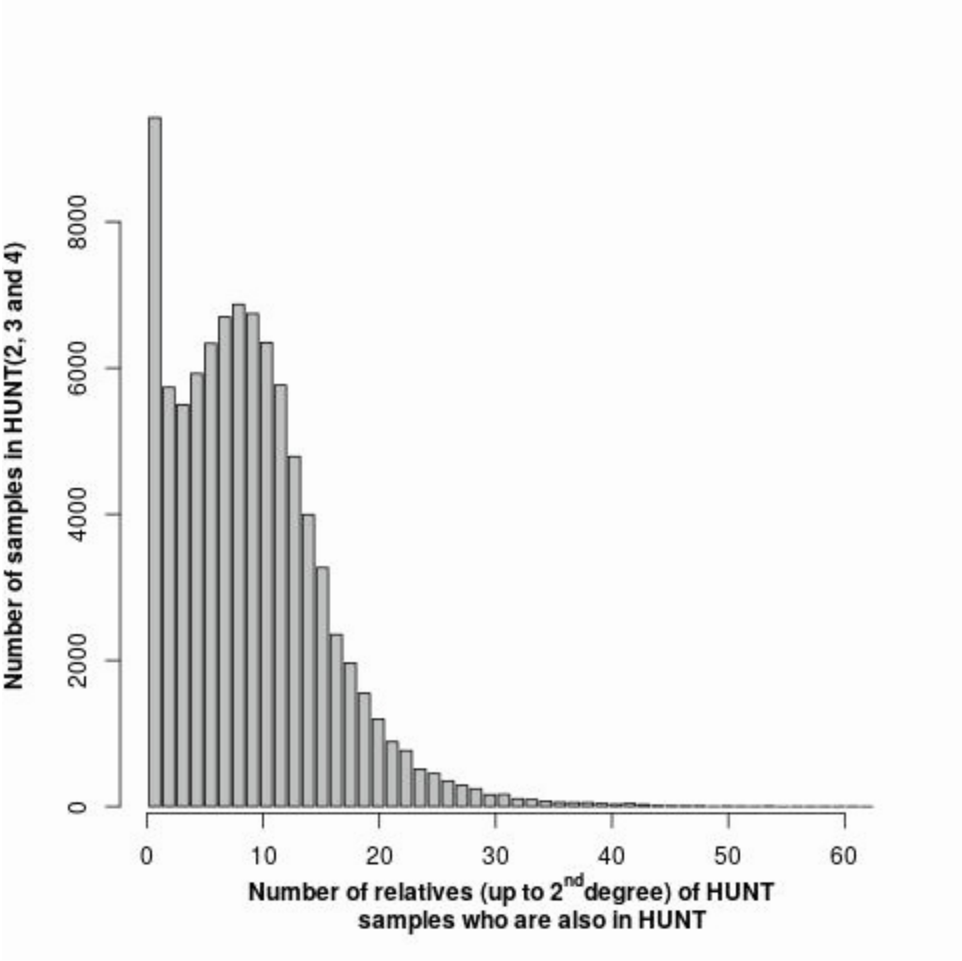

Figure S3: Histogram of numbers of up to 2nd degree relatives of HUNT2-4 samples who are also in HUNT (N=88 615). Related to Table 3.

HUNT: Trøndelag Health Study

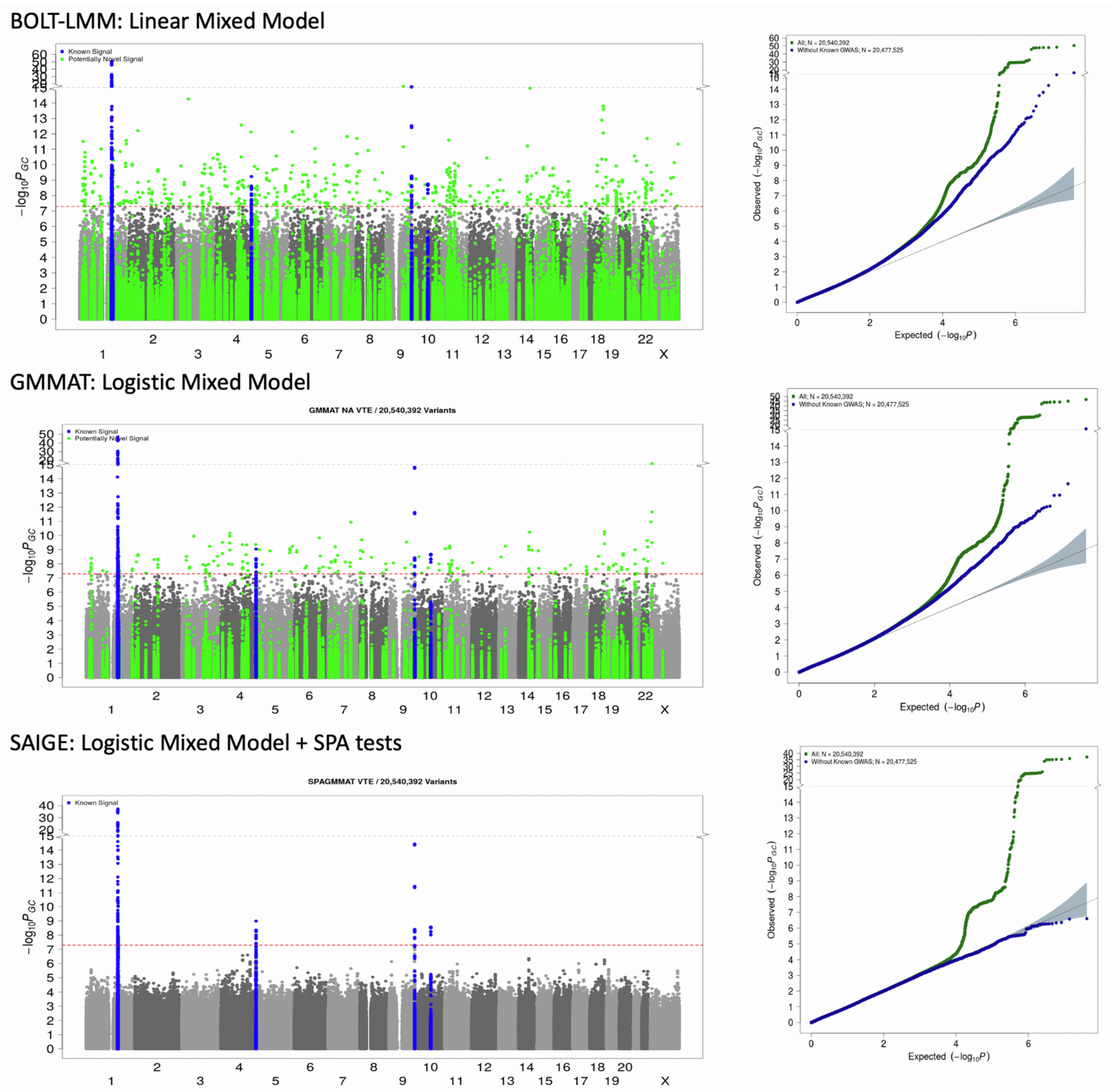

Figure S4: Analysis of venous thromboembolism (2 325 cases, 65 294 controls, case:control=0.036) in HUNT2-3 samples using BOLT-LMM, GMMAT and SAIGE (to account for relatedness and control for unbalanced case-control imbalance) of binary phenotypes. Related to Table 3.

HUNT: Trøndelag Health Study

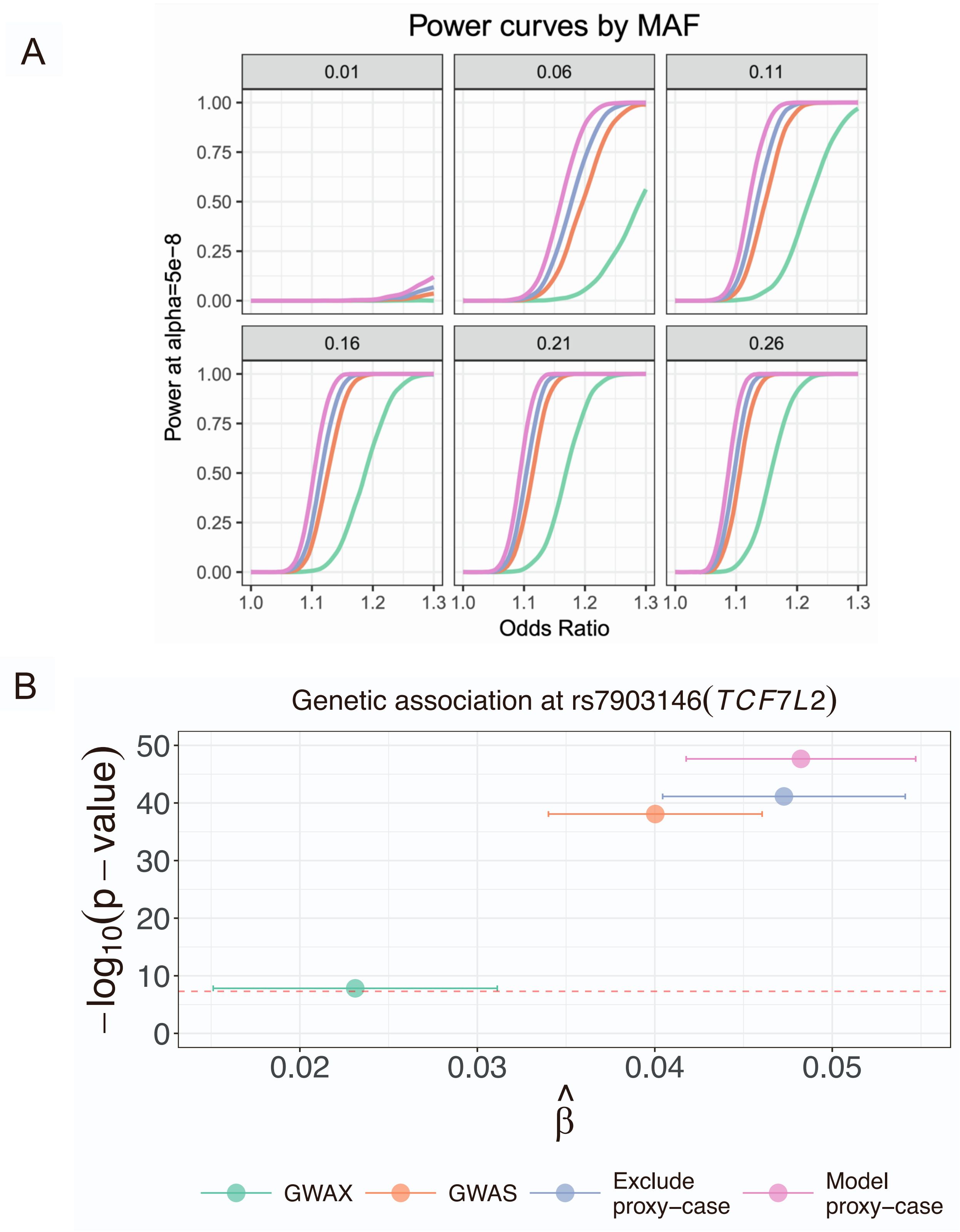

Figure S5: Power and empirical results for GWAS by proxy. Related to Table 3.

Panel A: Simulated power curves for proxy-case models. Given a total biobank size of 100,000 for a disease prevalence of 10% and heritability of disease liability of 10% a biobank could have cases (n=10,000),

proxy-cases (n=16,814) and controls (n=73,186). By performing linear regression with various methods of including proxy-cases, we can estimate the power at genome wide significance (p-value < 5e-8) across minor allele frequencies (0.01 to 0.26) and odds ratios (1 to 1.3). GWAX uses only proxy-cases as cases[1], GWAS is standard cases versus controls without identifying proxy-cases within the controls, excluding proxy-cases removes these for cleaner controls, and modelling proxy-cases uses coefficient of kinship for controls (F=0), proxy-cases (F=0.5), and cases (F=1) respectively. **Panel B: Association of the genetic variant rs7903146 with type 2 diabetes in HUNT across proxy-case models.** Empirical effect size and p-value for association at known type 2 diabetes variant rs7903146 in TCF7L2 in HUNT across methods of modelling proxy-cases. Linear mixed model as implemented in BOLT-LMM was used for 5.382 type 2 diabetes cases, 4,747 proxy-cases, and 20,284 controls. Proxy-cases were identified using self-reported first-degree family history of diabetes from HUNT questionnaires.

GWAS: Genome-wide association study, GWAX: Genome-wide association study by proxy, HUNT: Trøndelag Health Study, MAF: Minor allele frequency.

Supplementary Table 1: Summary of the variants in the HUNT whole-genome sequencing reference panel containing 2,201 individuals with average sequencing depth 5x. Related to STAR Methods (Imputation).

Variant Type	Total number of variants	Mean number of variants per individual (SD)	Mean number of unique variants per individual (SD)	% in 1000 Genomes	Number of novel variants*
**Splice**	1,265	71.5(4.6)	0.2(0.47)	36.6	355
**Nonsense**	2,432	71.5(6)	0.43(0.74)	36.6	585
**Missense**	113,576	9,480(113)	13.8(13.6)	56.3	13,927
**Synonymous**	77,699	10,707(100)	7.1(7.5)	68.5	5,935
**Noncoding**	20,050,237	3,342,839(15,415)	1,531(906)	68.7	4,030,199
**Total**	20,245,209	3,363,168(15,522)	1,552(919)	68.6	4,051,001

*Novel: not reported in dbSNP 144[2], 1000 Genomes Phase 3[3], UK10K[4], ESP6500 (*W. NHLBI GO Exome Sequencing Project (ESP) Seattle*, 2013), or ExAC.r0.3[5]

ANNOVAR[6] was used for the annotation with the default setting. Splice variants are variants within 2-bp of a splicing junction.

HUNT: Trøndelag Health Study, SD: Standard deviation.

Table S2: Overview of mandatory national registries, other national and regional registries. Related to Table 2.
See downloadable spreadsheet

*Started in 2012, but data is derived from different sources and therefore dates may vary.

Table S3. Relationship inference of genotyped samples from HUNT2-4 (N=88 721)*. Related to Table 3.

**Inference**	**Monozygotic twins**	**Parent-offspring**	**Full-siblings**	**2nd degree**
**Number of pairs**	106	68124	44777	192318

*To provide a full overview of the relationships in HUNT we included 106 pairs of twins in this analysis. The twin with the highest genotyping call rate is kept for other analyses.

HUNT: Trøndelag Health Study

Table S4: Selection of variants for inclusion in custom content for the HUNT-Michigan Illumina Infinium Human CoreExome arrays. Related to STAR Methods (Genotyping Procedures).

**Selection criterion for custom content on the HUNT human core exome v1.0**	**Variants that had a Final Score# > 0.5 AND designed into array as custom content**	**Polymorphic variants in ~60,000 Norwegian individuals**
Identified from HUNT sequencing* AND missense and minor allele count = 1 AND observed in 12,000 ESP samples (https://genome.sph.umich.edu/wiki/Exome_Chip_Design)	5427	4843
Identified from HUNT sequencing* AND missense and minor allele count >= 2	9853	8941
Identified from HUNT sequencing* AND LoF and minor allele count >= 1	836	731
Identified from HUNT sequencing* AND p < 1x10-4 for lipids or MI	1072	1006
NHGRI GWAS catalog 5/21/2014 AND $p < 5x10^{-8}$	1139	1056
GIANT associated variants NOT in NHGRI GWAS catalog	246	184
96 candidate genes including ACMG56 – any codon that would theoretically exist to produce a LoF	32,868*	2548 **
Norwegian LDLR mutations from FH clinic	149	34
Ancestry	3354	3085
Neandertal	5322	4835
Pain	99	83
Total custom variants	60365	27346
**Additional selection criterion for custom content on the HUNT human core exome v2.0**	**Variants that had a Final Score# > 0.5 AND designed into array as custom content**	**Polymorphic variants in 18537 Norwegian individuals**
LoF variant poorly imputed from TOPMed	449	350
Associated with bone mineral density	3	3
Associated with depression and/or alcohol use disorder	20	20
Associated with liver function	7	6
Associated with psoriasis	10	8
Breast cancer risk variant	204	123
GWAS catalog variant	1692	760
Subtotal additional custom variants	2385	1270
**Total custom variants**	62750	28616

* 32,868 putative LoF variants were assayed with only one bead type. For 21,640 out of 32,868 putative LoF variants, the alternate allele that would cause a premature stop codon cannot be distinguished from a missense or synonymous change.

[#] Final Score: Final scores are based on a proprietary algorithm from Illumina where values can range from 0—1 with higher values reflecting the likelihood of success for a particular marker.

ESP: Exome sequencing project, FH: Familial hypercholesterolemia, GWAS: Genome-wide association study, HUNT: Trøndelag Health Study, LDLR: Low density lipoprotein receptor, LoF: Loss-of-function, MI: Myocardial infarction, NHGRI: National Human Genome Research Institute, TOPMed: Trans-Omics for Precision Medicine.

Data S1. Collaboration between the HUNT study at the Norwegian University of Science and Technology, Norway and the University of Michigan, USA. Related to Table 3.

HUNT-MI Leadership: Kristian Hveem, Cristen J. Willer, Oddgeir L. Holmen, Michael Boehnke, Gonçalo R. Abecasis, Bjorn Olav Åsvold, Ben M. Brumpton; **Scientific Advisory Committee:** Ele Zeggini, Mark Daly, Bjørn Pasternak; **HUNT Research Centre:** Jørn Søberg Fenstad, Anne Jorunn Vikdal, Marit Næss; **HUNT Cloud:** Oddgeir L. Holmen, Sandor Zeestraten, Tom Erik Røberg; **Data applications and registry linkages:** Maiken E. Gabrielsen, Anne Heidi Skogholt; **Low-pass whole sequencing genome bioinformatics and statistical analysis:** He Zhang, Hyun Min Kang, Jin Chen; **Array genotyping:** Sten Even Erlandsen, Vidar Beisvåg; **GWAS bioinformatics, QC, imputation and statistical analysis:** Wei Zhou, Jonas Nielsen, Lars G. Fritsche, Hyun Min Kang, Oddgeir L. Holmen, Laurent Thomas and Ben M. Brumpton; **CNV calling:** Ellen Schmidt, Ryan Mills; **Statistical methods development for analyzing HUNT data:** Wei Zhou, Shawn Lee, Hyun Min Kang; **Communications management:** Bethany Klunder.

References

1. Liu JZ, Erlich Y and Pickrell JK. Case-control association mapping by proxy using family history of disease. *Nat Genet*. 2017;49:325-331.
2. Sherry ST, Ward MH, Kholodov M, Baker J, Phan L, Smigielski EM and Sirotkin K. dbSNP: the NCBI database of genetic variation. *Nucleic Acids Res*. 2001;29:308-11.
3. Genomes Project C, Auton A, Brooks LD, Durbin RM, Garrison EP, Kang HM, et al. A global reference for human genetic variation. *Nature*. 2015;526:68-74.
4. Consortium UK, Walter K, Min JL, Huang J, Crooks L, Memari Y, et al. The UK10K project identifies rare variants in health and disease. *Nature*. 2015;526:82-90.
5. Lek M, Karczewski KJ, Minikel EV, Samocha KE, Banks E, Fennell T, et al. Analysis of protein-coding genetic variation in 60,706 humans. *Nature*. 2016;536:285-91.
6. Wang K, Li M and Hakonarson H. ANNOVAR: functional annotation of genetic variants from high-throughput sequencing data. *Nucleic Acids Res*. 2010;38:e164.
