# Supplementary material for: The HUNT study: A population-based cohort for genetic research
Source: Cell Genom. 2022 Oct 12;2(10):100193. doi: 10.1016/j.xgen.2022.100193 (PMC9903730; doi:10.1016/j.xgen.2022.100193)
Supplement: Table S2. Overview of mandatory national registries, other national and regional registries, related to Table 2 [file mmc2.pdf]

Cell Genomics

Transparent Peer Review Record

The HUNT Study: a population-based cohort for genetic research

Ben M. Brumpton[1,2,3*#§], Sarah Graham[4*], Ida Surakka[4*], Anne Heidi Skogholt[1], Mari Løset[1,5], Lars G. Fritsche[6], Brooke Wolford[7,8], Wei Zhou[9,10], Jonas Bille Nielsen[11], Oddgeir L. Holmen[1,2], Maiken E. Gabrielsen[1,2], Laurent Thomas[1,12,13], Laxmi Bhatta[1], Humaira Rasheed[1], He Zhang [4], Hyun Min Kang [6], Whitney Hornsby [4], Marta Riise Moksnes[1], Eivind Coward[1], Mads Melbye[1], Guro F. Giskeødegård[1], Jørn Fenstad[2], Steinar Krokstad[2,14], Marit Næss[2,14], Arnulf Langhammer[2,14], Michael Boehnke[6], Gonçalo R. Abecasis[15], Bjørn Olav Åsvold[1,2,16], Kristian Hveem[1,2*#], Cristen J. Willer[1,4,6,17*#]

Summary

Initial submission: Received : 12/16/2021

Scientific editors: Judith Nicholson, Laura Zahn

First round of review: Number of reviewers: 2
Revision invited : 3/10/2022
Revision received : 5/9/2022

Second round of review: Number of reviewers: N/A
Accepted : 9/13/2022

Data freely available: Requires registration

Code freely available:N/A

This transparent peer review record is not systematically proofread, type-set, or edited. Special characters, formatting, and equations may fail to render properly. Standard procedural text within the editor's letters has been deleted for the sake of brevity, but all official correspondence specific to the manuscript has been preserved.

Referees' reports, first round of review

Reviwer#1

The HUNT Study: a population-based cohort for genetic research
Brumpton BM et al.

This overview paper of the HUNT study is generally well-written. They have clearly maximised genotyping efforts by direct genotyping and imputation using different reference panels. In particular, a good justification is made as to the usefulness of using participants with a high degree of relatedness for genetic research and the analytical methods that have been developed to account for this.

I have some comments and suggestions for clarification that I hope are not too onerous.

Main comments

1. It would be helpful if the authors could make a stronger case for having genetic data in a wide range of populations - i.e. what does having genetic data that is specific to the Norwegian population (as opposed to other populations) enable researchers to do?

2. This paper focuses on the use of HUNT as a resource for genetic analyses, which begs the question why is a prospective study design required (given the high cost and long duration of a longitudinal study). Can the authors comment on how the study has (or can) facilitate prospective research? For example, they state that they have intentionally repeated questionnaire items to assess change in measures - why is this important?

Minor points

3. Fig 1. Legend should say county (not country)

4. Can you state what measures (if any) were taken to ensure the laboratory (and other) measures are of high quality - e.g., measured with tight QC, measures at one time point in one lab using one device, etc.?

5. There is no mention of socio-economic status. While you state that the cohort is representative of the general population, is it possible to include in Table 1 more socio-economic/demographic variables? Even in Norway, there must be some socioeconomic inequality that is important to capture for epidemiological purposes.

6. Can you add a statement about consent - specifically for the linkage to health and health-related datasets?

7. Can you explicitly comment on whether you have linkage to primary care (GP records)?

8. Table 2. It would be worth stating in the text that these nos. are based on hospital data and so are likely to result in under-ascertainment of less-serious common conditions (asthma, diabetes, depression, migraine, etc.). It would also be useful to comment on the usefulness of incident numbers (as opposed to total numbers that are provided in the table) for prospective research (see point 2).

9. Given the highly collaborative nature of genetic epidemiology, can you state what proportion of researchers accessing the data are not affiliated with HUNT, so the reader can get a feel for how widespread data access really is?

Reviewer#2

This cohort profile focuses on the genomic aspects of the Trøndelag Health Study, a large cohort with a number of unique features which distinguish it from other biobanks, including its extended longitudinal follow-up with low loss-to-follow-up and the nature of the population included. The manuscript includes an overview of the phenotypic data, and describes a range of methodologies developed for genetic studies in the context of studies with high relatedness. The manuscript also briefly cites GWAS and meta-analyses relating to a wide range of phenotypes to which the resource has contributed. The resource will have broad utility and be of interest to researchers in many fields.

Major comments

1. It would aid completeness to mention key limitations of the study that are particularly relevant to genomic studies. For example, given that this is a general population cohort, limitations might include limited power for GWAS of uncommon phenotypes, particularly if using a traditional two-stage design. This would support the argument for collaboration and "team science" on page 19. Another consideration might be generalisability of findings to populations of non-European ancestry and the importance of replicating findings elsewhere.
2. I assume that the requirement for international researchers to collaborate with a Norwegian principal investigator is in place to facilitate application to the regional ethics committee. How does the study balance this with principles of openness of access and ensuring that the potential utility of the resource is fulfilled? Are there particular arrangements to support/facilitate access for researchers from low- and middle-income countries, for example?

Minor comments

1. Pages 7-8: A flowchart of the genotyping and imputation of HUNT2-3 would aid the reader, either as part of Figure 4 or a supplementary figure.
2. Table 1: Given that the measurements are presented at the last study attendance, it would provide useful context to present age at last study attendance in addition to first study attendance.
3. Page 12: It is not clear whether the linkage to non-health-related registries is already established or a future development, or that there is some additional complexity with data access? I note that such registries are included in Supplementary Table 2. I also note that in the data access section, the process for accessing health-related registries is described, but the process for non-health-related registries is not. Please clarify on page 12 and in the data access section.
4. Page 15: There is a reference to SAIGE missing.
5. Page 15: The abbreviation GLGC should be defined.
6. Supplementary Table 4: Design Score should be defined.

Authors' response to the first round of review

We thank the reviewers for their time and the opportunity to revise and resubmit our manuscript. We have carefully considered the valuable comments raised by the reviewers and address their comments below.

Reviewer #1: The HUNT Study: a population-based cohort for genetic research
Brumpton BM et al.

This overview paper of the HUNT study is generally well-written. They have clearly maximised genotyping efforts by direct genotyping and imputation using different reference panels. In particular, a good justification is made as to the usefulness of using participants with a high degree of relatedness for genetic research and the analytical methods that have been developed to account for this.

I have some comments and suggestions for clarification that I hope are not too onerous.
Main comments

1. It would be helpful if the authors could make a stronger case for having genetic data in a wide range of populations - i.e. what does having genetic data that is specific to the Norwegian population (as opposed to other populations) enable researchers to do?

Thank you for the comment. We agree that genetic data should be ascertained from a wide range of populations. A key strength of the HUNT study is due to the uniqueness of the population where; 1) we found population-specific variants enriched in HUNT [1]; 2) we were able to use up to 40 years of existing longitudinal biomedical research data, including data seldom measured elsewhere [2] and, 3) most adult inhabitants participated [2], providing some protection from selection bias, and allowing opportunities for family-based designs [3]. Collecting genetic data in a wide range of populations would have similar strengths that could provide complementary resources for research.

To make a stronger case for having genetic data both in Norway and a wide range of populations, we have highlighted these strengths in the summary section (page 17, paragraph 3).

"We hope that initiatives such as this, which capture population-specific variants, use up to 40 years of existing longitudinal biomedical research data and where the majority of adult inhabitants participated, make a strong case for why it is important to have genetic data both in Norway and a wide range of populations."

2. This paper focuses on the use of HUNT as a resource for genetic analyses, which begs the question why is a prospective study design required (given the high cost and long duration of a longitudinal study). Can the authors comment on how the study has (or can) facilitate prospective research? For example, they state that they have intentionally repeated questionnaire items to assess change in measures - why is this important?

We agree with the reviewer that it could be seen as an inefficient design for genetic discovery, however the data can be used to predict disease and there are now upcoming methods which can be used to understand disease progression and survival where we hope HUNT will be a major contributor. GWAS to-date have generally focused on disease onset, but it is not given that risk factors will influence both disease onset and disease progression. Therefore, GWAS on traits related to disease progression, such as change in lung function or depression scores among those with COPD or Major Depression, respectively, may directly translate into the identification of potential therapeutic targets. Repeated questionnaires and measurements for this type of analyses are available in HUNT.

To make this point clear, we have highlighted this in the phenotypes section (page 7, paragraph 2).

"Importantly, many measurements and questionnaire items have been intentionally kept identical or similar across HUNT surveys to enable longitudinal analyses, which may contribute to understanding disease progression and survival."

Finally, we are also very fortunate that the establishment of the epidemiological data in HUNT was established before this genetic initiative, providing a long legacy of data at no additional expense.

Minor points
3. Fig 1. Legend should say county (not country)

Thank you. Corrected

4. Can you state what measures (if any) were taken to ensure the laboratory (and other) measures are of high quality - e.g., measured with tight QC, measures at one time point in one lab using one device, etc.?

To ensure data was of high quality, biologic material was handled at the field stations according to appropriate standards and transported to the biobank every evening in a cold chain. Several measurements including hemoglobin and blood cell counts, creatinine and cholesterol were sent for immediate analysis and were performed by specially trained personnel according to the same standardized protocols with the same equipment. Both plasma, serum and buffy coat are stored in aliquots in automated freezers in HUNT Biobank at -80°C. The databank website describes each measure in more detail including specific details of the instrument used and coefficients of variation (https://www.ntnu.edu/hunt/databank).

In addition to phenotypic laboratory measurements, genotypic measurements followed a strict protocol based upon the approach developed by the Johns Hopkins Center for Inherited Disease Research (CIDR) and that of Guo et al[4]. This included excluding samples and variants that failed to reach a 99% call rate, resulting in genotyping 358 964 polymorphic variants.

We now include a statement describing quality assurances of the phenotypic laboratory measurements in the phenotypes section (page 7, paragraph 2) and genotypic quality assurances in the measurements in "Genotyping and imputation study design in HUNT" section (page 5, paragraph 1).

"To ensure data was of high quality, biologic material was handled at the field stations according to appropriate standards and transported to the biobank every evening in a cold chain. Several measurements including hemoglobin and blood cell counts, creatinine and cholesterol were sent for immediate analysis and were performed by specially trained personnel according to the same standardized protocols with the same equipment. Both plasma, serum and buffy coat are stored in aliquots in automated freezers in HUNT Biobank at -80°C. The databank website describes each measure in more detail including specific details of the instrument used and coefficients of variation (https://www.ntnu.edu/hunt/databank)."

"We followed a strict quality control protocol based upon the approach developed by the Johns Hopkins Center for Inherited Disease Research (CIDR) and that of Guo et al[4]. This included excluding samples and variants that failed to reach a 99% call rate, resulting in genotyping 358 964 polymorphic variants."

5. There is no mention of socio-economic status. While you state that the cohort is representative of the general population, is it possible to include in Table 1 more socioeconomic/ demographic variables? Even in Norway, there must be some socioeconomic inequality that is important to capture for epidemiological purposes.

We have added education attainment and household income to Table 1.

6. Can you add a statement about consent - specifically for the linkage to health and health-related datasets?

We now included a statement about consent (page 18, paragraph 2) and linkage to health and health related datasets in the manuscript (page 10, paragraph 1).

The respective sections now read:

"HUNT participants have consented to linkage to the many high-quality health and administrative registries in Norway, and to information from medical records."

"The genotyping in HUNT and work presented in this cohort profile was approved by the Regional Committee for Ethics in Medical Research, Central Norway (2014/144, 2018/1622, 152023). All participants signed informed consent for participation and the use of data in research."

7. Can you explicitly comment on whether you have linkage to primary care (GP records)? Linkage to primary care is available from the Norwegian Registry for Primary Health Care and Norwegian Control and Payment of Health Reimbursements Database. We have now added this to the section on Linkage to regional and national health registries (page 10, paragraph 1), which now reads:

"Commonly used national registries linked with HUNT include the Norwegian Cause of Death Registry (established 1951), the Cancer Registry of Norway (established 1952), the Medical Birth Registry of Norway (established 1967), the Norwegian Prescription Database (established 2004), Norwegian Control and Payment of Health Reimbursements Database (established 2006) and the Norwegian Registry for Primary Health Care (established 2017)"

8. Table 2. It would be worth stating in the text that these nos. are based on hospital data and so are likely to result in under-ascertainment of less-serious common conditions (asthma, diabetes, depression, migraine, etc.). It would also be useful to comment on the usefulness of incident numbers (as opposed to total numbers that are provided in the table) for prospective research (see point 2).

We have added the comment "Numbers based on hospital data are likely to result in underascertainment of less-serious common conditions." to the footnote of Table 2. Due to the multiple surveys conducted in HUNT we have not presented incident numbers. However, we have now highlighted an empirical example which used 2,974 incident coronary artery disease

cases from after HUNT2 in the "Linkage to regional and national health registries" section (page 11, paragraph 1)5.

9. Given the highly collaborative nature of genetic epidemiology, can you state what proportion of researchers accessing the data are not affiliated with HUNT, so the reader can get a feel for how widespread data access really is?

Approximately half of the researchers in this collaboration with data access are not affiliated with NTNU. We have added a statement to highlight active research collaborations at non-NTNU campuses in the Contribution to collaborative studies section (page 18, paragraph 2):

"These contributions highlight efforts from researchers in equal parts from the K.G. Jebsen Center for Genetic Epidemiology, NTNU, Norway and the University of Michigan Medical School and the University of Michigan School of Public Health, USA."

Reviewer #2: This cohort profile focuses on the genomic aspects of the Trøndelag Health Study, a large cohort with a number of unique features which distinguish it from other biobanks, including its extended longitudinal follow-up with low loss-to-follow-up and the nature of the population included. The manuscript includes an overview of the phenotypic data, and describes a range of methodologies developed for genetic studies in the context of studies with high relatedness. The manuscript also briefly cites GWAS and meta-analyses relating to a wide range of phenotypes to which the resource has contributed. The resource will have broad utility and be of interest to researchers in many fields.

Major comments
1. It would aid completeness to mention key limitations of the study that are particularly relevant to genomic studies. For example, given that this is a general population cohort, limitations might include limited power for GWAS of uncommon phenotypes, particularly if using a traditional two-stage design. This would support the argument for collaboration and "team science" on page 19. Another consideration might be generalisability of findings to populations of non-European ancestry and the importance of replicating findings elsewhere.

Thank you for the comment. We have now included limitations relevant to genomic studies in the manuscript (see below - page 18, paragraph 2).

"While the HUNT study has been an essential cohort in the genetic discoveries and causal inference mentioned so far, used in isolation it is limited due to low power to investigate uncommon phenotypes, uncertainty of the generalizability of findings to non-Europeans, and the lack of an independent sample for replication. To overcome these limitations, we contribute to genetic studies worldwide through participation in consortia focused on a variety of diseases"

2. I assume that the requirement for international researchers to collaborate with a Norwegian principal investigator is in place to facilitate application to the regional ethics committee. How does the study balance this with principles of openness of access and ensuring that the potential utility of the resource is fulfilled? Are there particular arrangements to support/facilitate access for researchers from low- and middle-income countries, for example?

Your understanding that medical research in Norway is governed by Norwegian law is correct. In some part we believe that this has contributed to the high participation rates that we see in Norway. Participants also have the expectation that their data will be used and there are no barriers to whom can collaborate. Regarding data access there are no special arrangements for low- and middle-income countries. However, the fees are kept to a minimum for all to cover administrative costs and do not represent a source of revenue that maintains or builds the cohort.

Minor comments
1. Pages 7-8: A flowchart of the genotyping and imputation of HUNT2-3 would aid the reader, either as part of Figure 4 or a supplementary figure.

We have added a second panel to Figure 4 to clarify the genotyping and imputation approach (see below).

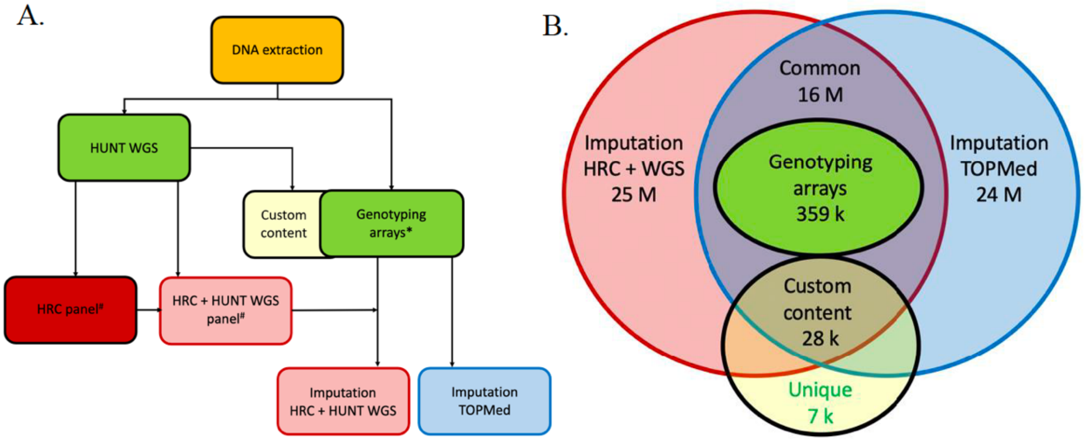

Figure 4: Genotyping and imputation-based approach in HUNT.
Panel A. Flowchart of the approach.
Panel B. Number of variants captured by each approach.
HUNT: Trøndelag Health Study, HRC: Haplotype Reference Consortium, k: Thousand, M: Million, TOPMed: Trans-Omics for Precision Medicine, WGS: Whole-genome sequencing.
**Not all genotyping arrays included custom content*
#Only 1,200 HUNT samples were sequenced at the time the HRC was established, however 2,201 were included in the HRC + HUNT WGS panel.

2. Table 1: Given that the measurements are presented at the last study attendance, it would provide useful context to present age at last study attendance in addition to first study attendance.

We have added age of last attendance to Table 1.

3. Page 12: It is not clear whether the linkage to non-health-related registries is already established or a future development, or that there is some additional complexity with data access? I note that such registries are included in Supplementary Table 2. I also note that in the data access section, the process for accessing health-related registries is described, but the process for non-health-related registries is not. Please clarify on page 12 and in the data access section.

Thank you for pointing this out. We now include at the beginning of the paragraph that participants have consented to both linkage of health and non-health related registries (Linkage to regional and national health registries section, page 10, paragraph 1). We have also changed the term used for "non-health related registries" to "administrative registries" to describe the content of the registers more precisely. We have made corresponding changes in the data access section (page 19, paragraph 1):

"Data linkages between HUNT and health or administrative registries require that the principal investigator has obtained project-specific approval for such linkage from the Regional Committee for Medical and Health Research Ethics, Norway and each registry owner."

4. Page 15: There is a reference to SAIGE missing.

Added.

5. Page 15: The abbreviation GLGC should be defined.

Corrected.

6. Supplementary Table 4: Design Score should be defined.

Thank you for pointing this out. To make the term consistent with Illumina headers we have now changed this to "Final Score" and include the definition in the footnote:

"Final scores are based on a proprietary algorithm from Illumina where values can range from 0—1 with higher values reflecting the likelihood of successful genotyping of the particular marker."

References

1. Surakka, I. et al. MEPE loss-of-function variant associates with decreased bone mineral density and increased fracture risk. Nat Commun 11, 4093 (2020).

2. Åsvold, B.O. et al. Cohort Profile Update: The HUNT Study, Norway. medRxiv, 2021.10.12.21264858 (2021).

3. Brumpton, B. et al. Avoiding dynastic, assortative mating, and population stratification biases in Mendelian randomization through within-family analyses.

Nature communications 11, 1-13 (2020).

4. Guo, Y. et al. Illumina human exome genotyping array clustering and quality control. Nat Protoc 9, 2643-62 (2014).

5. Surakka, I. et al. Sex-specific survival bias and interaction modeling in coronary artery disease risk prediction. medRxiv, 2021.06.23.21259247 (2021).
